# Phenotyping transcription factors-related genotypes in *Y. lipolytica* across a range of industrially relevant process parameters and chemical stress conditions

**DOI:** 10.1186/s12934-026-02986-z

**Published:** 2026-03-17

**Authors:** Maria Gorczyca, Abinaya M. G. Ponmalar, Julia Matz, Jean-Marc Nicaud, Ewelina Celińska

**Affiliations:** 1 Department of Biotechnology and Food Microbiology, Poznan University of Life Sciences, ul. Wojska Polskiego 48, 60-637 Poznań, Poland; 2 AgroParisTech, Université Paris-Saclay, INRAE, Micalis Institute, 48 rue Pablo Picasso, 78350 Jouy-en- Josas, France; 3 Yalicolor, 48 rue Pablo Picasso, 78190 Trappes, France

**Keywords:** *Yarrowia lipolytica*, Transcription factor, Global metabolic engineering, Stress resistance, Toxic compounds, Environmental stress

## Abstract

One of the factors contributing to “the death valley” in biotech process transfer is an assumption that once a synthetic trait is successfully introduced and optimized in a microbial host, the resulting strain attains “industrial potential”. In practice, most engineered strains lack the robustness required to perform sufficiently in the demanding conditions of industrial bioprocesses. Stress tolerance is a complex, non‑pathway trait that is tightly regulated and highly polygenic, making it challenging to engineer. Consequently, beyond constructing a desired phenotype, substantial effort must also be directed toward improving the overall “robustness” of engineered strains. In this work, we systematically phenotyped transcription factor (TF)–related genotypes of Yarrowia lipolytica under a broad spectrum of industrially relevant stress conditions. Eight TFs were chosen based on prior high‑throughput screening results (YaliFunTome database). For each TF, two additional genotypes—overexpression (OE) and knockout (KO)—were generated to enable a comprehensive assessment of TF function. All three genotypes per TF were evaluated for growth under diverse stress exposures, including process parameters (oxygen availability, pH, and temperature) and a range of toxic chemicals relevant to industrial processes: lignocellulosic biomass-derived inhibitors, short‑chain fatty acids, osmotic inducers, salt, mild acid, and oxidative stressors, antifungal agent, and alcohols. This study delivers new insights—or deepens existing understanding—of how selected TFs contribute to process parameters and chemical stress resistance in Y. lipolytica. Based on these findings, we propose global metabolic engineering strategies to enhance strain robustness under industrial stress conditions. These include Msn4‑, Hap1‑OE, and Yas1‑KO for improved thermotolerance; Yas1‑OE for enhanced osmotic stress resistance; Dal81‑OE and KO of TF011 or Mhy1 for increased tolerance to lactic acid; and Yas1‑OE or KO of TF011 and Mhy1 for improved growth in the presence of the valuable aroma and antifungal compound 2‑phenylethanol (2PE).

**Supplementary Information:**

The online version contains supplementary material available at 10.1186/s12934-026-02986-z.

## Introduction

The microbial biotechnology “promise” is that once a synthetic trait is established and optimized in a microbe, the strain reaches “industrial potential”, as can be read in multiple papers. Yet, the “problem” is that in the majority of cases, the strains are not robust enough to withstand the harsh industrial conditions. Stress resistance is a non-pathway trait, tightly regulated and very difficult to engineer due to its multigenic nature [1–4]. Hence, apart from establishing a targeted phenotype, efforts towards enhancing the “robustness” of an engineered strain are undertaken. To this end, researchers reach for “global (metabolic) engineering”. Technically, microbial robustness can be improved by adaptive laboratory evolution [5, 6], using polyploid and/or industrial strains as engineering platforms [7, 8], longevity engineering [9], synthetic induction of stress response via genetic [10–12] or bioprocessing methods [13–15], enhancing global translation activity [16], using “buffer” genes [17] or chromatin remodelers [18, 19], and others.

In our recent studies, we were interested in global transcription machinery engineering, a strategy first coined in yeast research by [1, 20]. The idea is to use global transcriptional regulators (transcription factors or chromatin remodelers) to modulate whole regulomes, as they were evolved by nature. This way, by a relatively limited number of genetic interventions, massive changes are induced. Importantly, these regulomes evolved to serve a specific purpose, i.e., defense against a specific or multiple environmental stressors or to attenuate internal imbalances [21, 22]. Hence, the elicited response is both targeted and comprehensive, covering all the necessary, even non-intuitively associated, biological functions. We focus our studies on *Yarrowia lipolytica*, a yeast species of increasing industrial interest [23, 24]. The first high-throughput global transcription machinery engineering was conducted in this species for enhanced lipid accumulation [25–27]. Afterwards, studies on the intensification of recombinant protein (rProt) biosynthesis under various conditions followed [28]. Results of these high-throughput phenotyping screens can be browsed in an open database, YaliFunTome (https://sparrow.up.poznan.pl/tsdatabase/). Yet, while highly comprehensive (covering over 100 TFs each), all those studies relied on strains bearing overexpression (OE) of an individual transcription factor (TF) and phenotyping growth, rProt, or lipid synthesis level. Those high-throughput overexpression screens provided rapid information on the beneficial impact of the TFs’ high-level OE (pTEF promoter [29, 30]) on the trait of interest. TF-engineering in *Y. lipolytica* for globally adjusted phenotypes covering complete reverse engineering (OE and knock-out; KO) was first conducted on a small scale [31] for four TFs (*HSF1*-YALI0E13948g, *GZF1*-YALI0D20482g, *CRF1*-YALI0B08206g, *SKN7*-YALI0D14520g). Only recently, a high-throughput TFs’ expression tuning in *Y. lipolytica* (from KO to high-level OE) was employed to engineer thermotolerance, morphotype, and production of betanin [32]. It relied on cloning and managing libraries of clones in mixtures (7 promoters x 56 TFs), followed by high-throughput screening to rapidly fish out the desired phenotypes. With a strong focus on the applicatory outcome, the desired phenotypes were efficiently and rapidly determined, but the insight into the TFs’ characteristics was less elaborated.

In this study, we aimed to enhance the resistance of *Y. lipolytica* towards multiple industrially relevant stress conditions, leveraging a global transcriptional machinery engineering and complete reverse engineering (OE vs. KO). To this end, we selected eight TFs (Table 1) that, when co-overexpressed with rProt ([28], YaliFunTome database), displayed relevant phenotypes, such as enhanced/abolished growth under limited oxygen availability (OA) or low pH. The selected TFs were both KO and OE in the background without additional rProt overexpression. The idea was to gain a comprehensive view of the actual TF’s involvement in a particular, industrially relevant phenotype. As mentioned above, the previous insight into a specific TF function in *Y. lipolytica* was limited to interrogation of a single genotype (KO or OE). Here, three genotypes were used to characterize the TF’s function (OE, KO, and OE with additional rProt). Furthermore, the generated library of strains was then phenotyped across a wide range of conditions, covering process parameters stress factors (combinations of OA, pH, temperature) and exposure to toxic chemicals, relevant to multiple industrial processes. These included toxic products of lignocellulosic biomass pre-treatment, short-chain fatty acids, inducers of hyperosmolarity, salt, acid, oxidative stress, antifungal, and alcohols. While limited in the number of TFs compared to the previous reports on *Y. lipolytica* TFs, this study provides a comprehensive characterization of the TFs preselected in the high-throughput screens. Universal regularities, specific responses, as well as inverted phenotypes (OE vs. KO), suggesting a direct implication of a gene in the phenotype under study, were identified.

**Table 1:** List of TF genes studied. Custom name (if available) and reference to Yali0 and Yali1 version of the genome are given

Gene	Yali0 number	Yali1 number	YaliFunTome results (OE)	Putative function	InterPro domain search
Dal81	YALI0D02783g	YALI1_D03561g	https://sparrow.up.poznan.pl/tsdatabase/?page=gene&name=TF118	nitrogen degradation pathways, involved in nitrogen catabolite activation; glucose transport regulator (Rgt1-related)	Zn(2)-C6 fungal-type
Hap1	YALI0F17424g	YALI1_F23191g	https://sparrow.up.poznan.pl/tsdatabase/?page=gene&name=TF120	oxygen sensing and signaling	Zn(2)-C6 fungal-type
Mhy1	YALI0B21582g	YALI1_B28150g	https://sparrow.up.poznan.pl/tsdatabase/?page=gene&name=TF095	Mns2/Mns4-like protein, a key regulator of yeast-to-hypha dimorphic transition but not stress resistance, regulates both alkaline-pH and glucose-induced filamentation	Zinc finger C2H2
Msn4	YALI0C13750g	YALI1_C19151g	https://sparrow.up.poznan.pl/tsdatabase/?page=gene&name=TF107	The general stress response regulates tolerance to acid-induced stress and antioxidant cellular response	Zinc finger C2H2
Jmc2	YALI0B14443g	YALI1_B18975g	https://sparrow.up.poznan.pl/tsdatabase/?page=gene&name=TF009	histone demethylase, promotes global demethylation of H_3_K_4_	JmjC
TF011	YALI0B20944g	YALI1_B27360g	https://sparrow.up.poznan.pl/tsdatabase/?page=gene&name=TF011	Enhance growth in low OA	Zn(2)-C6 fungal-type
Lac9	YALI0D20460g	YALI1_D25968g	https://sparrow.up.poznan.pl/tsdatabase/?page=gene&name=TF036	induction of the lactose-galactose regulation	Zn(2)-C6 fungal-type
Yas1	YALI0C02387g	YALI1_C03349g	https://sparrow.up.poznan.pl/tsdatabase/?page=gene&name=TF083	essential for cytochrome p450 induction in response to alkanes, heteromeric Yas1p/Yas2p complex transcription factor/ similar to S. cerevisiae INO4 TF required for derepression of inositol-choline-regulated genes involved in phospholipid synthesis	Basic HLH

Links direct to specific pages in YaliFunTome database showing previous results on the TF’s phenotyping

## Materials and methods

### Microbial strains and routine maintenance

All the *Y. lipolytica* strains used in this study are derivatives of either Po1f (*MatA*,* leu2-270*,* ura3-302*,* xpr2-322*,* axp-2*; phenotype: ΔAEP, ΔAXP, ura-, leu-; ATCC MYA-2613) or JMY2810 (genotype: *MATa*,* ura3::pTEF-RedStar2-LEU2-Zeta-URA3ex-pTEF-empty*,* leu2-270*,* xpr2-322*; phenotype: ΔAEP, ΔAXP, suc+, ura+, leu+, intracellular RedStar2, Zeta platform; [25]). The overall set comprised: (i) 16 newly constructed strains (8 bearing TF OE and 8 with TF KO, each containing a single modification in the respective TF *locus*); (ii) 8 strains co-overexpressing (co-OE) a given TF together with inRedStar2 (Leplat et al., 2015); and a respective control strain for each subset. *Escherichia coli* was used for vector subcloning. The full list of strains is given in Tables S1 and S2.

*Y. lipolytica* and *E. coli* strains were maintained according to standard protocols [33, 34]. *E. coli* DH5α and its derivatives were grown at 37 °C with 200 rpm shaking in LB medium (g/L: yeast extract, 5 (Biomaxima, Poland), bacteriological peptone, 10 (Biocorp, Poland), NaCl, 5 (PoCh, Gliwice, Poland)), supplemented with kanamycin (40 µg/mL) or ampicillin (100 µg/mL), and agar, 15 (BTL, Poland), when required. Yeast strains were routinely maintained in YNB medium (g/L: YNB w/o amino acids (AA) and ammonium sulfate (AS) 1.7 (Merck-Millipore), ammonium sulfate 5 (PoCh), glucose 20 (PoCh)), or in YPD medium (g/L: yeast extract 10, bacteriological peptone, 20, glucose, 20), solidified with agar, 15, when required, at 28 °C. Liquid cultures were run in 24-square-well plates and shaken at 250 rpm.

### Molecular biology techniques and reagents

*E. coli* and *Y. lipolytica* transformations followed standard protocols, heat-shock and lithium acetate heat-shock, respectively, as described in [33, 34]. Restriction endonucleases (*AvrII*, *BamHI*, *NotI*, *EcoRI*, *KpnI;* Thermo Fisher Scientific, Waltham, Massachusetts, USA), Phire DNA polymerase (Thermo Fisher Scientific), T4 DNA ligase (New England Biolabs, Ipswich, USA), and pCR Blunt II TOPO vector (Thermo Fisher Scientific) were all used according to protocols provided by the respective manufacturers. DNA plasmid isolation, DNA fragment extraction from agarose gel and purification were performed with corresponding kits from A&A Biotechnology (Gdynia, Poland).

The TFs modified in this study are listed in Table 1. Full DNA sequences of the TFs to be OE (codon-optimized, synthetic versions) are listed in Table S3. All the oligonucleotides used for amplification of the TFs, or homologous arms from genomic DNA, are listed in Table S4.

### Cloning strategy

The background strain was constructed by transforming the Po1f strain with the URA3 cassette (JMP62-URA3ex; *ura + leu-*). This was then either transformed with the LEU2 cassette (JMP62-LEU2ex; *ura + leu+*; to generate a background strain for deletions (KO strains) or a control strain), or with a JMP62-LEU2ex bearing a respective TF sequence (OE strains). All the DNA fragments were first subcloned into a pCR Blunt II TOPO vector to verify the sequence.

#### TF overexpression – OE strains

The TF genes were either directly amplified from genomic DNA or ordered from GeneCust (Boynes, France) as synthetic DNA fragments in the pUC57 vector. Genes *MSN4*, *JMC2*, *DAL81*, and *YAS1* were amplified with specific flanks *BamHI* at 5’ and *AvrII* at 3’ ends added. *MHY1*, *TF011*, *LAC9*, and *HAP1* were purchased as chemically synthetized DNA fragments, due to internal *BamHI* recognition sites, further codon-optimized, and with the respective overhangs at the termini. After subcloning into the pCR Blunt II TOPO vector, the TF-encoding fragment was cloned into JMP62-LEU2ex under the control of the constitutive pTEF promoter.

Bacterial parts of the plasmids were removed prior to transformation by digestion with the *NotI* restriction enzyme. The transformations were run in subsequent rounds to increase the effectiveness and number of correct clones. Firstly, the parental strain Po1f was transformed with an empty JMP62-URA3ex cassette, followed by plating on YNB Drop-Out medium (Drop-Out without uracil; Y1501, Merck-Millipore). The second round of transformation was run with JMP62-LEU2ex-pTEF-TF and plated onto plating on YNB Drop-Out medium (Drop-Out without histidine, leucine, tryptophan, and uracil; Y2001, Merck-Millipore). At least 12 clones (up to 24) were picked after 2 days of incubation at 28 °C for screening.

#### TF deletion – KO strains

The KO cassettes were constructed on the backbone of the pV2 vector [35]. The scheme is shown in Figure S1. First, the nourseothricin resistance gene (NATr) was cloned between the *KpnI* and *BamHI* recognition sites to generate the pV2-NATr vector. The homologous arms flanking the NATr gene were amplified from the Po1f strain genomic DNA. Around 1000 bp were amplified upstream/downstream ATG/stop codon, respectively (taking care not to interfere with adjacent ORFs). Upstream homologous arm (ArmUP) was flanked with *EcoRI* and *KpnI*, and the downstream arm (ArmDown) with *BamHI* and *NotI*, recognition sites at 5’ and 3’ ends, respectively. The individual fragments for homologous arms were first subcloned into the pCR Blunt II TOPO vector and further cloned into pV2-NATr. The correct assembly of expression cassettes was verified by PCR, enzyme digestion, and sequenced (Genomed, Warsaw, Poland). The ArmUP-NATr-ArmDown cassette was released from pV2-NATr by digestion with *EcoRI* and *NotI* endonucleases and transformed into Y. *lipolytica* strain (Po1f derivative with ura + leu+). The transformants were plated onto YPD NAT (100 µg/ml) medium and incubated at 28 °C. At least 5 clones were picked after 2–3 days of incubation at 28 °C for screening.

All the strains were deposited as 15% glycerol stocks at -80 °C.

### Phenotyping – stress-inflicting batch cultures

All the cultivations were run in 24-well Duetz-System square plates in 2.5 mL working volume (EnzyScreen BV, Netherlands), agitated at 250 rpm in a LabCompanion shaker-incubator (SANLAB, Poland). The cultures were continued for 72 h, and samples were collected at 3 time-points, every 24 h. Precultures were developed for 18 h at 28 °C. The main cultures were inoculated at 4% (v/v).

#### Process parameters stress

Three process parameters stress factors were tested in combinations: oxygen availability (OA; “+” denotes 2.5 mL exchanged air/min; “-“ denotes 0.004 mL exchanged air/min; EnzyScreen BV, Netherlands [36]), pH (3.0 vs. 5.0), and temperature (28°C vs. 34°C). Maleic acid (PoCh) buffer at 0.1 or 0.2 M concentration was used to stabilize the pH at pH 3.0 and 5.0, respectively [28, 37, 38]. The basic medium used in these tests was YPD. Altogether, 8 treatment combinations were applied.

#### Chemical stress

Ten chemical compounds, known to induce different types of cellular stress, were tested in this study. These were (g/L): Congo Red (CR), 0.2, Menadione (Men), 5 mM, lactic acid (LA), 30, propionic acid (PA), 3, 2-phenylethanol (2PE), 1.5, vanillin (Van), 1.5, sorbitol (Sorb), 364.34, NaCl, 102.27, Furfural (Fur), 1, 5-hydroxymethylfurfural (5HMF), 4. Since 2PE, Van, Fur, and 5HMF had to be dissolved in ethanol, ethanol at 40 g/L was also used as an additional control. All the concentrations were first carefully pre-optimized for each of the control strains (Po1f-derived prototroph and JMY2800), so that they cause at least 50% growth limitation at 48 h under the adopted cultivation conditions. The control culture was run in 3xYNB medium (g/L): YNB w/o amino acids (AA) and ammonium sulfate (AS) 5.1 (Merck-Millipore), ammonium sulfate 7.5 (PoCh), glutamic acid, 7.5 (Merck-Millipore), glucose 20 (PoCh), maleic acid, 23.2 (PoCh), and NaOH (PoCh) to pH 5.0.

#### Sample collection and analytical methods

Samples were collected at 0, 24, 48, and 72 h of cultivation. Samples were diluted in 0.75% NaCl to fit into the linear range of the absorbance measurement (absorbance at 600 nm wavelength; < 1.0). Measurements were run in a 96-well flat-bottom transparent MTP plate at 600 nm in the Tecan Spark automatic plate reader (Tecan Group Ltd., Mannedorf, Switzerland).

### Data processing and statistical analysis

Representative subclones for each genotype were selected from the subclones screening cultures using a custom Python script. With the use of clustering algorithms the clones with the predominant phenotype were selected.

Growth data from the stress-inflicting batch cultures were processed with a custom, automated Docker-based pipeline, to calculate growth, generate visual summaries, and perform statistical testing.

Statistical significance of differences between modified strains and control, as well as “inverted phenotype” for the corresponding strains, were assessed for each condition and time point using one-way ANOVA followed by Tukey post-hoc testing, with a significance threshold of *p* ≤ 0.05. All the “inverted phenotypes” mentioned hereafter are statistically significant, the selected results are gathered in Table S5. Relative change (RC) in growth was calculated by subtracting the growth of the control strain (in corresponding stress conditions) from the studied TF and dividing by the growth of the control strain. The container ensures fully reproducible and standardized analysis from raw microplate outputs to final statistics and figures. All the analyses were performed in Python 3.11.9.

Re-processing of public RNA-seq data (“data-recycling”). RNA-seq datasets relevant to the stress factors investigated in this study were retrieved from the NCBI Sequence Read Archive (SRA) (Table S6). Raw sequencing data were downloaded with SRA Toolkit (NCBI SRA-Tools. https://github.com/ncbi/sra-tools, Accessed December 2025), followed by adapter trimming and quality filtering (Cutadapt [39]), and quality control (FastQC; [40]) using a wrapper tool Trim Galore! (Trim Galore. https://github.com/FelixKrueger/TrimGalore, Accessed December 2025); summary quality reports were compiled with MultiQC [41]. Libraries that failed quality control were excluded from further analysis. Reads were aligned to the reference genome (strain W29 – assembly ASM176148v1) with STAR [42], with single-end and paired-end libraries processed according to their layout. Library strandedness was inferred using the RSeQC tool infer_experiment.py [43]. Gene-level read counts were obtained with featureCounts [44] in exon mode, using parameters appropriate for the inferred strandedness and read layout. RNA-seq count data were imported and normalized using the edgeR package [45, 46]. Differential expression analysis was performed using the limma framework [47]. Principal component analysis was conducted on the transcriptomic data and visualized using ggplot2. All steps (download, trimming, alignment, counting, and DEG analysis) were orchestrated with custom Bash scripts, and analyses were performed using Python 3.11.9 and R 4.1.2.

## Results

### TFs modulating the process parameters stress resistance in *Y. lipolytica*

**Fig. 1:**
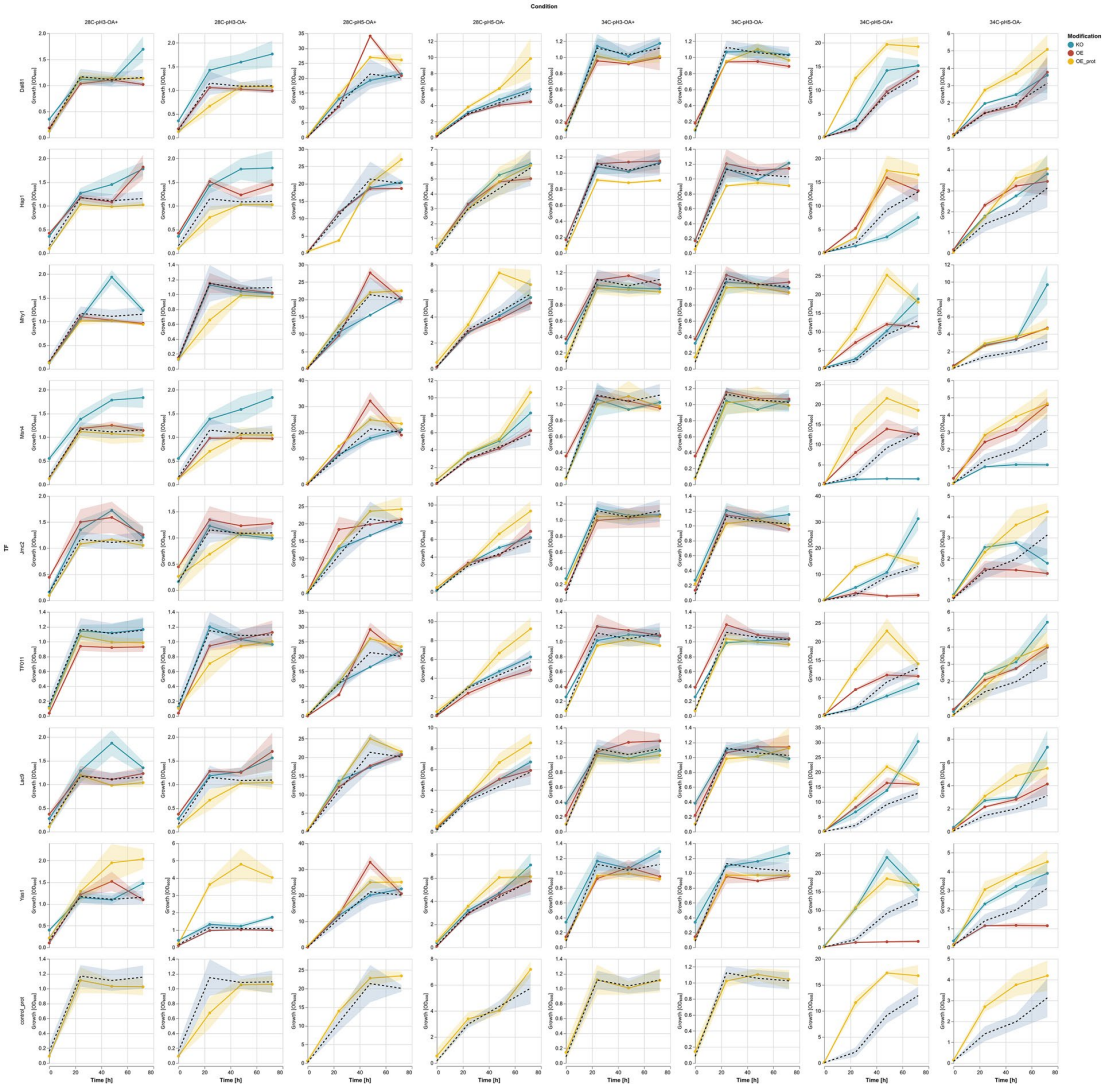
Growth of TF-modified and the control *Y. lipolytica* strains in process parameters stress factor-perturbed cultures. X axis: time [h]. Y axis: OD600 units. Y axes are adjusted to the OD600 readout for better visualization of the growth curves. Rows correspond to different TFs; columns - to different treatment conditions. Curves are colored according to the given legend. Shaded areas along the curves correspond to the ± SD from at least quadruplicate

**Fig. 2:**
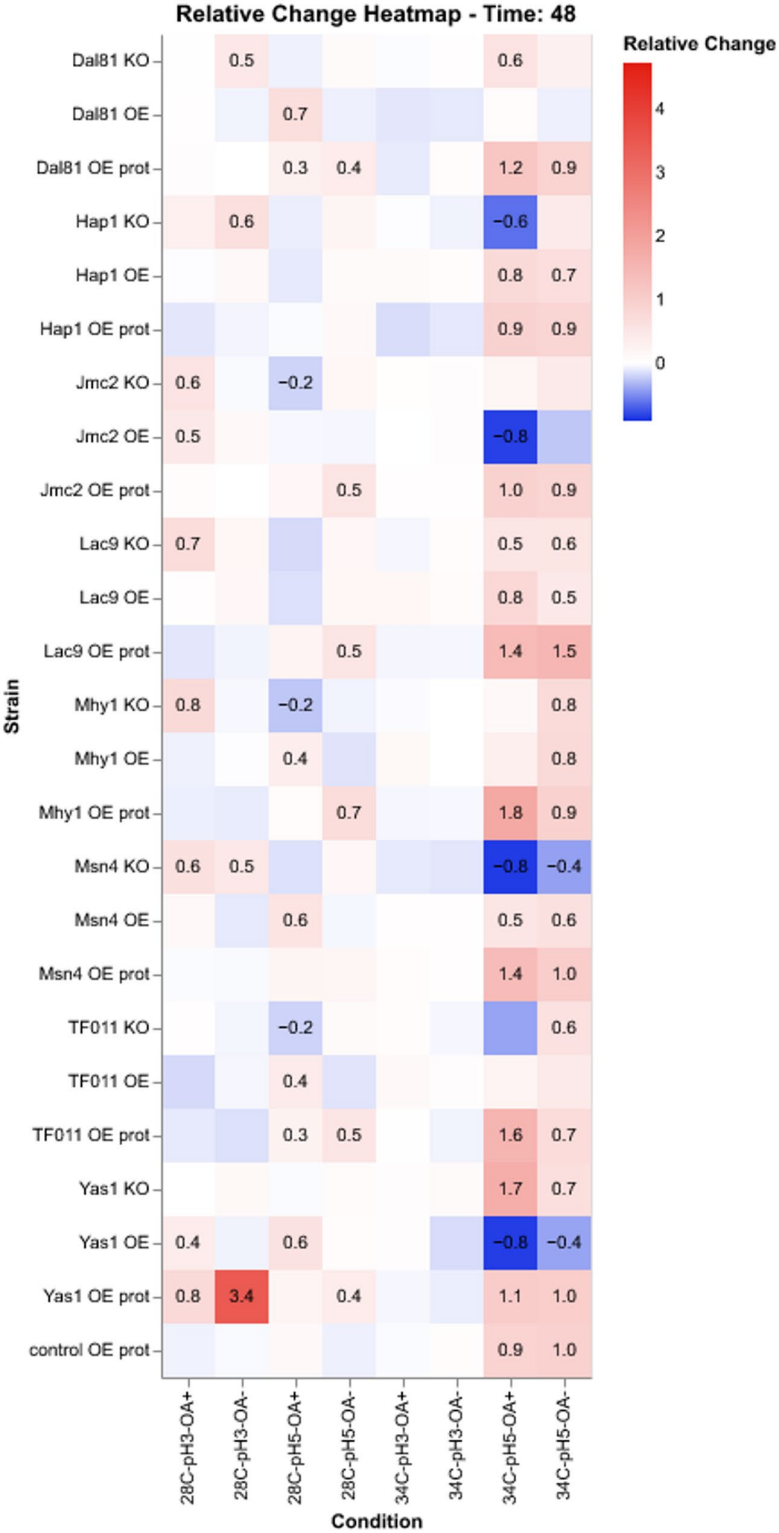
Relative changes in growth of TF-modified and the control *Y. lipolytica* strains in process parameters stress factor-perturbed cultures in the form of a heatmap—data acquired at 48 h of culturing. X axis: treatment conditions; Y axis: TF-modified strains. Squares at the cross-points show the RC value for a specific strain and condition. Numbers are given only for statistically significant RC. The color code is explained in a legend

**Fig. 3:**
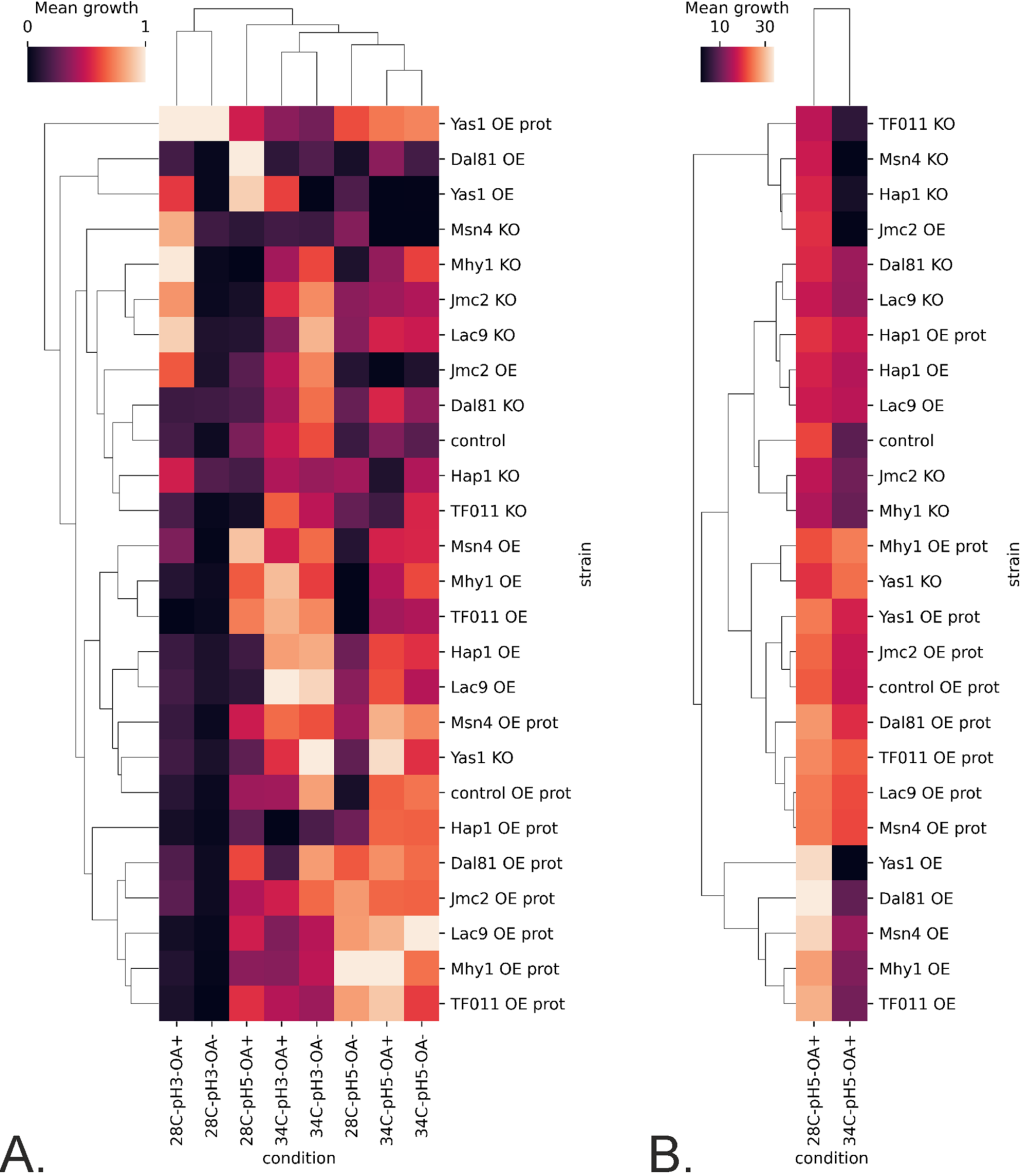
Clustered heatmap with a hierarchical dendrogram of normalized growth profiles of TF-modified and the control *Y. lipolytica* strains in process parameters stress factor-perturbed —data acquired at 48 h of culturing. **A**. All the conditions were considered, and the data were normalized within each condition. **B** Only thermal treatment (34 °C) vs. the corresponding control is considered; data were not normalized. X axis: treatment conditions; Y axis: TF-modified strains. Squares at the cross-points show the mean value of growth (standardized or not) for a specific strain and condition. Color code is explained in the legend

Figure 1 presents the growth results from the TF-modified and control strains cultured under the infliction of the process parameters stress (OA, pH, and temperature) throughout the cultivation time. Since the 48 h time point was found to be the most representative for the cultures run according to the adopted protocol [28, 37], Figs. 2 and 3 present further analyses conducted on data from this time point. Figure 2 is a heatmap illustrating the relative change in growth (RC; as defined above) of the TF-modified strains compared to the control strain, across various combinations of conditions. Figure 3A, B presents the results of hierarchical clustering analysis in the form of a dendrogram, which groups the most similar conditions and strains.

Considering the inflicted conditions, pH 3.0 severely limited the growth of both background strains (control and control OE prot; Fig. 1, bottom row). Hierarchical clustering facilitated a global view and comparison of the results (Fig. 3A, B). Condition-wise normalization and grouping (Fig. 3A) revealed the prevalent impact of pH 3.0, followed by temperature (28 vs. 34 °C). At a pH of 3.0, the limiting impact of oxygen deprivation was not observed (Fig. 3A). Only under growth-permissive pH (5.0), the detrimental impact of the “OA-“ condition was observable, limiting growth by 2- to 4-fold (Fig. 1; column 3 vs. 4, and 7 vs. 8; Fig. 3A). However, OA had a minor impact (conditions OA + and OA– were grouped together). The control condition (28°C, pH 5.0, OA+) formed a cluster of its own. The sole temperature upshift created another distinct condition (Fig. 3A), highly differentiating the genotypes. At a temperature of 34°C and a pH of 3.0, the growth curves were completely flattened, disallowing any genotype-driven phenotype manifestation at a significant level (Fig. 1, columns 5 and 6; Fig. 2); the combination of 34°C and pH 3.0 was clustered together, irrespective of OA (Fig. 3A). Hereafter, the combination of 34°C/pH 3.0 was not considered, as it disallows phenotype development.

Condition-wise analysis of the strains’ growth at 48 h (Fig. 3A), highlighted the genotype Yas1-OE prot as the most resistant to pH downshift and relatively resistant to temperature upshift. Interestingly, a series of KO strains (in Msn4, Mhy1, Jmc2, Lac9, Hap1) grew better at a pH of 3.0 than the control (Figs. 2 and 3A). Condition-wise normalization highlighted that under OA- (28 °C, pH 5.0; Fig. 3A), the majority of strains bearing the extra burden of the rProt over-synthesis and a TF (OE prot) grew better than the average (valid for Yas1, Dal81, Jmc2, Lac9, Mhy1, TF011). It was not valid only for the Hap1 and Msn4 OE in the “OE prot” background, yet they still grew better than the control.

At the permissive temperature (28 °C), the growth curves at pH 3.0 were more differentiated than under the combination of pH 3.0 and 34 °C (Fig. 1, columns 1 and 2), and the impact of specific TF-engineering could be observed (Fig. 2). Notably, Yas1-OE(prot) strains displayed significantly higher growth compared to the control (Figs. 1, 2 and 3). In addition, Yas1-modified strains showed consistent responses at elevated temperature (34 °C) and the permissive pH (5.0). Under these conditions, an inverted phenotype was observed between the Yas1 OE (down RC) and KO (up RC) strains (Fig. 2). Genotype Yas1 OE prot escaped this notion – even though Yas1 was OE, the strain displayed significant enhancement in growth, indicating a significant role of the background rProt synthesis on the strain’s growth.

The temperature of 34 °C turned out to be the condition that differentiated the TF-modified strains the most (Figs. 2 and 3B). Both Hap1-OE and Hap1-OE prot strains displayed elevated growth as opposed to the KO strain. In this regard, Hap1 was similar to Msn4; OE of Msn4 (either with or without an extra OE in the background) triggered a significant increase in growth under 34 °C, while its KO led to the opposite effect. Under 34 °C, a uniform enhancement in growth was observed consistently for all the strains bearing the background OE of a reporter protein, including the control strain. The majority of these strains were clustered together (Fig. 3B), demonstrating a universal character of the effect, independent of the TF-modification. As seen in Figs. 1 and 3B, sole OE of rProt enhanced growth of the strain under 34 °C (control vs. control OE prot) (Fig. 2). Considering the clustering analysis (Fig. 3B), the OE prot genotype acted as a “buffer”, mitigating the effect of the interplay between the condition and the TF-modification (seen for OE and KO strains, without rProt).

For Jcm2 and Yas1, sole TF OE caused a downshift in growth under 34 °C, while when combined with the protein overproduction, the effect was inverted (Fig. 2). In contrast, the Yas1-KO promoted growth, but Jcm2-KO did not affect growth under 34 °C when compared to the control conditions (Figs. 2 and 3B). A similar lack of effect of KO under 34 °C was also observed for Mhy1, indicating no direct impact of the TF modification under these conditions. Similar to Jcm2, the temperature of 34 °C triggered the severe RC decreases driven by KO of TF011, Msn4, and Hap1 (Figs. 2 and 3B); all these strains were clustered together in a distinct cluster with a unique profile.

The strains OE of Yas1, Dal81, Msn4, Mhy1, and TF011 (Fig. 3B) displayed a remarkable phenotype change between the two thermal conditions – at 28 °C, they strongly promoted growth vs. the control, at 34 °C, the growth was either reduced or comparable to the control. In general, OE of Dal81 and TF011, Mhy1, Msn4, and Yas1 promoted the growth of the *Y. lipolytica* strains when no stress treatment was applied. The phenotype was inverted when the genes were KO (Fig. 1, column 3; Figs. 2 and 3A; Table S5).

As mentioned above, the impact of the limited oxygen availability (OA- vs. OA+) can be reliably assessed only for conditions at a pH of 5.0. Then, under the most favorable combination of conditions (based on Figs. 1 and 3B °C, pH 5.0), the growth under OA- was enhanced for Mhy1, Jmc2, Lac9, and Yas1 OE prot. That was not valid when an extra high-temperature treatment was applied (the difference OA + vs. OA- was non-significant); yet growth was always higher when oxygen was in excess (Fig. 2). A combination of 34 °C and OA- conditions revealed an interesting effect for TF011 modification. Its KO caused limited growth under full oxygen provision, while enhanced (34 °C) or maintained at the background level (28 °C) under oxygen deprivation. The opposite trends were observed under TF011 OE. No such effect was observed when rProt was additionally co-overexpressed.

### TFs modulating the chemical stress resistance in *Y. lipolytica*

**Fig. 4:**
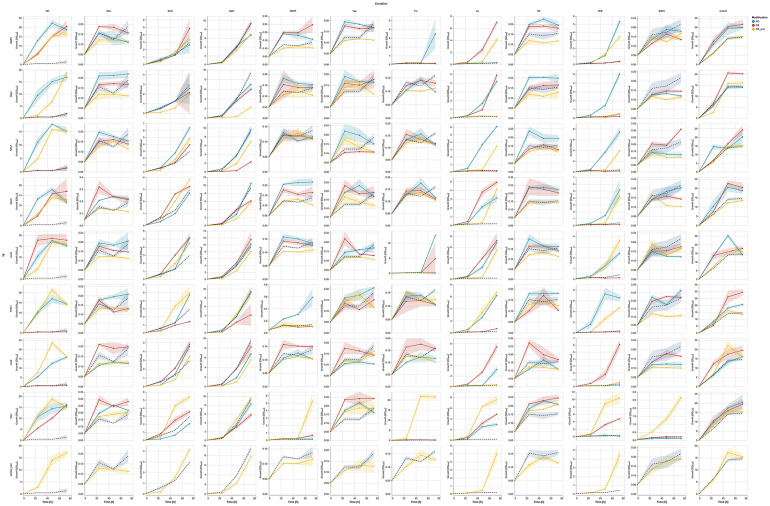
Growth of TF-modified and the control *Y. lipolytica* strains in chemical stress factor-perturbed cultures. X axis: time [h]. Y axis: OD600 units. Y axes are adjusted to the OD600 readout for better visualization of the growth curves. Rows correspond to different TFs; columns - to different treatment conditions. Curves are colored according to the given legend. Shaded areas along the curves correspond to the ± SD from at least quadruplicate

**Fig. 5:**
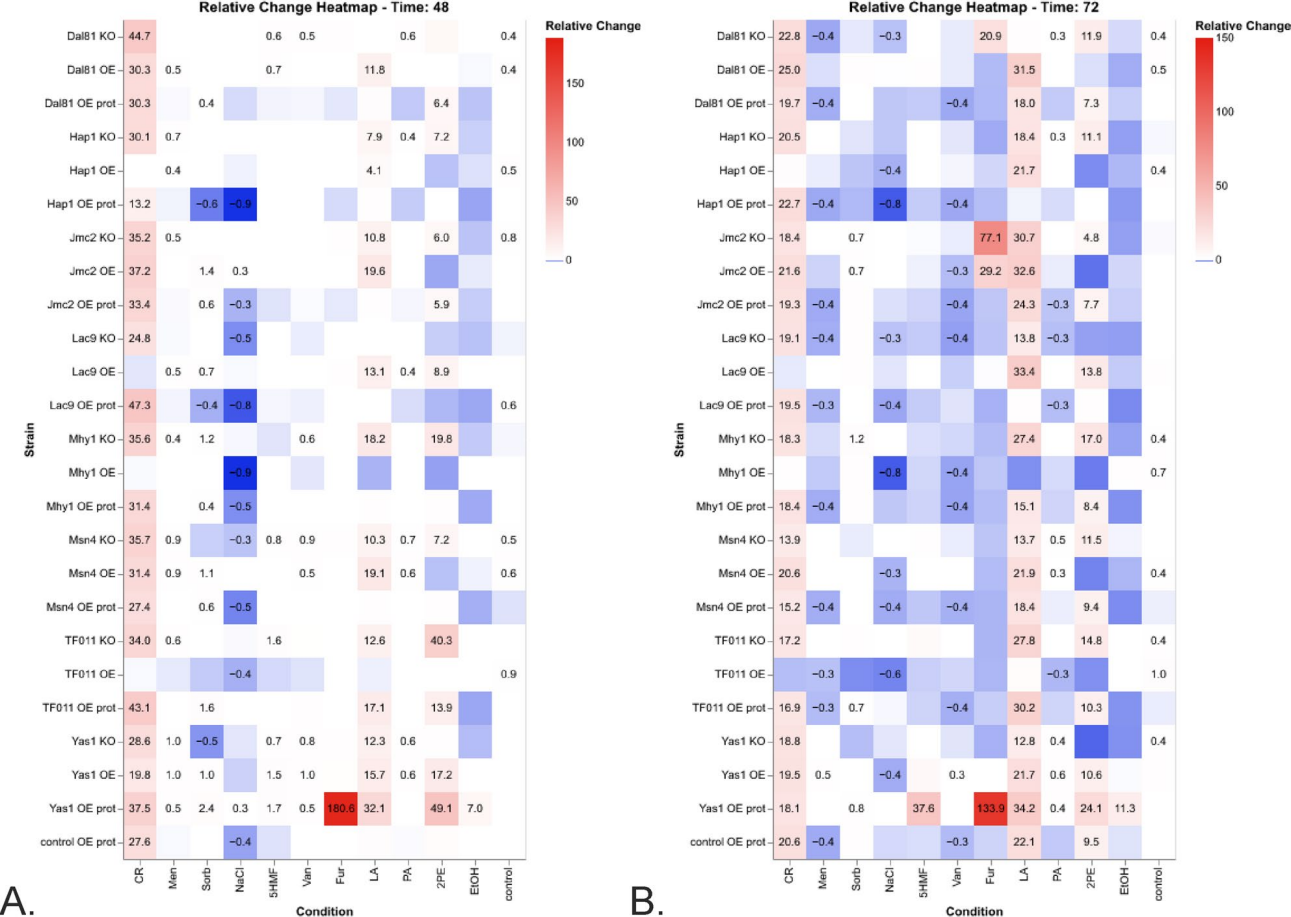
Relative changes in growth of TF-modified and the control *Y. lipolytica* strains in chemical stress factor-perturbed cultures in the form of a heatmap—data acquired at 48 h **A** and 72 h **B** of culturing. X axis: treatment conditions; Y axis: TF-modified strains. Squares at the cross-points show the RC value for a specific strain and condition. Numbers are given only for statistically significant RC. The color code is explained in a legend

In line with the data for process parameters stress resistance, Fig. 4 presents the growth of the TF-modified and the control strains cultured in media containing toxic compounds at pre-defined limiting concentrations (see Materials and Methods; CR, Men, LA, PA, EtOH, 2PE, Van, Sorb, NaCl, Fur, 5HMF, and the control medium) throughout the cultivation time. Figures 5 (heatmap based on RC) and 6 (hierarchical clustering) present further analyses conducted on data from 48 h to 72 h.

**Fig. 6:**
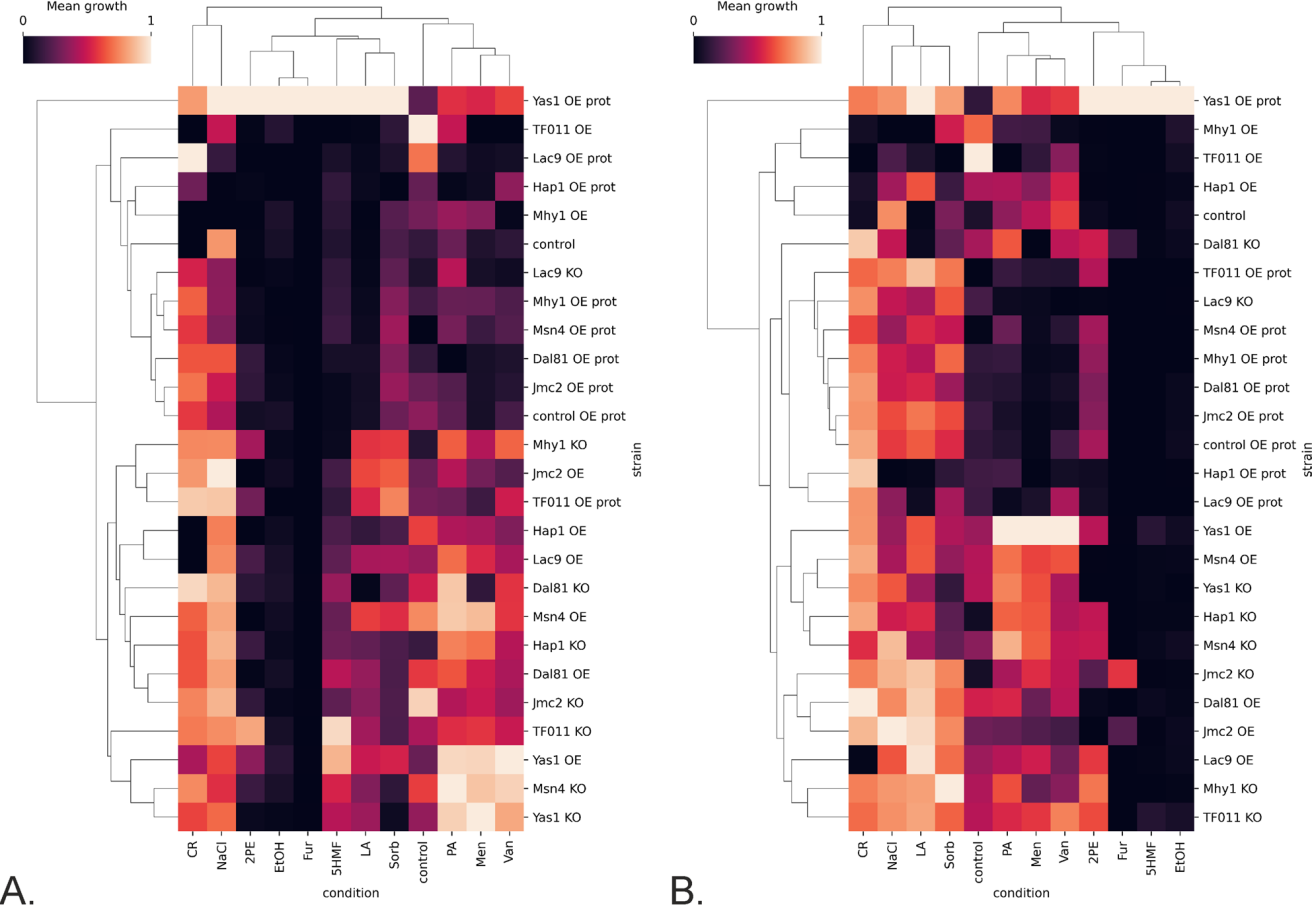
Clustered heatmap with a hierarchical dendrogram of normalized growth profiles of TF-modified and the control *Y. lipolytica* strains in process parameters stress factor-perturbed —data acquired at **A** 48 h and **B** 72 h of culturing. All the data were normalized within each condition. X axis: treatment conditions; Y axis: TF-modified strains. Squares at the cross-points show the standardized value of mean growth for a specific strain and condition. Color code is explained in the legend

Based on the control strains’ growth presented in Fig. 4 (bottom row), we concluded that all the conditions applied were set adequately for the experiment’s aim. Conditions: Men, 5HMF, Van, Fur, PA, and EtOH were the most limiting for the control strains; LA and 2PE enabled delayed growth; the osmo-stress inducers (Sorb and NaCl – extra salt stress) facilitated steady but limited growth. Even for the OE prot control strain grown in CR medium, which displayed increased growth compared to the control strain, the growth was still lower than in the control conditions (Fig. 4, bottom row). The most (EtOH, 5HMF, and Fur) and the least (CR and NaCl) limiting conditions were clustered together based on the growth pattern triggered across the collection of strains (Fig. 6A, B).

The majority of strains (either KO, OE, or OE prot) grew significantly better than the control in CR medium, but strains with OE of Hap1, Lac9, Mhy1, and TF011 displayed complete growth abolition (Figs. 4 and 5A, B). The oxidative stress infliction (Men) triggered an inverted phenotype for Lac9- and Dal81-modified strains (OE increased RC), and for TF011 (KO increased RC) (Figs. 4 and 5A; Table S5). Under the condition NaCl, multiple OE strains displayed decreased growth vs. the control strain (Fig. 5A; similar tendency for EtOH). In the following time point (Fig. 5B), a significant drop in RC in growth was observed for the majority of strains and conditions (tendencies and significant downshifts). Several interesting exceptions were identified. Apart from the positive RC for the TF-modified strains grown in CR, we observed that the majority of strains acquired resistance to LA. Also, all the analyzed TF displayed inverted phenotypes/increased ratio OE vs. KO for resistance to 2PE (Yas1-OE, Lac9-OE, TF011-KO, Msn4-KO, Mhy1-KO, Jcm2-KO, Hap1-KO, Dal81-KO); but it was not reflected in clustering (Fig. 6AB; Table S5).

As can be read from the overall growth pattern (Fig. 4), several TF-modified strains displayed interesting growth behavior. As in the case of process parameters stress results (Figs. 1 and 2), the Yas1-OE prot strain outperformed the other strainsin terms of resistance to the majority of toxic compounds (Figs. 4 and 5A, B, 6A, B). The response was clearly condition-induced, as under control growth conditions, the strains Yas1-OE(prot) grew comparably to the controls and the KO strain. The most striking upshifts were observed in EtOH, 2PE, LA, Fur, CR, and Sorb. Strain Yas1-OE prot was the unique strain among the “OE prot” strains that displayed enhanced resistance towards NaCl. For all the other OE strains with an additional rProt OE, NaCl significantly limited growth (Fig. 5A). Similarly, sole Yas1 OE triggered significantly higher growth than the control strain under most of the studied conditions (Fig. 5A, B). An inverted phenotype for Yas1-engineered strains was observed under Sorb and 2PE provision. OE of Lac9 TF improved resistance to Men, Sorb, LA, PA, and 2PE. Some growth-promoting effect of this modification was observed when the strains were grown in lignocellulosic-biomass-derived inhibitors (Fig. 4). It was the only TF-OE modification that yielded such consistent results for this group of toxic compounds. Yet, the upshift was too small to be statistically significant (Fig. 5A, B). Likewise, Msn4-KO triggered a similar growth-promoting effect towards these compounds (Fig. 4), yet again, too small to make it significant (Fig. 5A, B). The Msn4-KO strain significantly enhanced resistance to 2PE, yielding an inverted phenotype when OE. The same was observed for the other general stress response TF, Mhy1. In addition, the Mhy1 KO strain displayed growth improvement in LA and Van, and Mhy1 OE-triggered sensitivity to NaCl.

Strikingly, while the TF011-driven phenotype was not particularly responsive to process parameters stresses, its KO was relevant to several toxicity-related conditions. We observed a highly consistent growth upshift in CR, LA, and 2PE-containing media, and Men and 5HMF, but to a lesser extent (Figs. 4 and 5A, B). On the other hand, the TF011-OE strain in the majority of conditions triggered decreased growth. Jcm2 was interesting for its growth-promoting effect when KO and grown in 2PE, displaying an inverted phenotype for OE.

Hap1 KO strain displayed significantly enhanced resistance to CR, Men, 2PE, as well as PA (Fig. 5A, B). No obvious phenotype was seen under Hap1-OE.

### Global patterns of TF-driven process parameters and chemical stress resistance

**Fig. 7:**
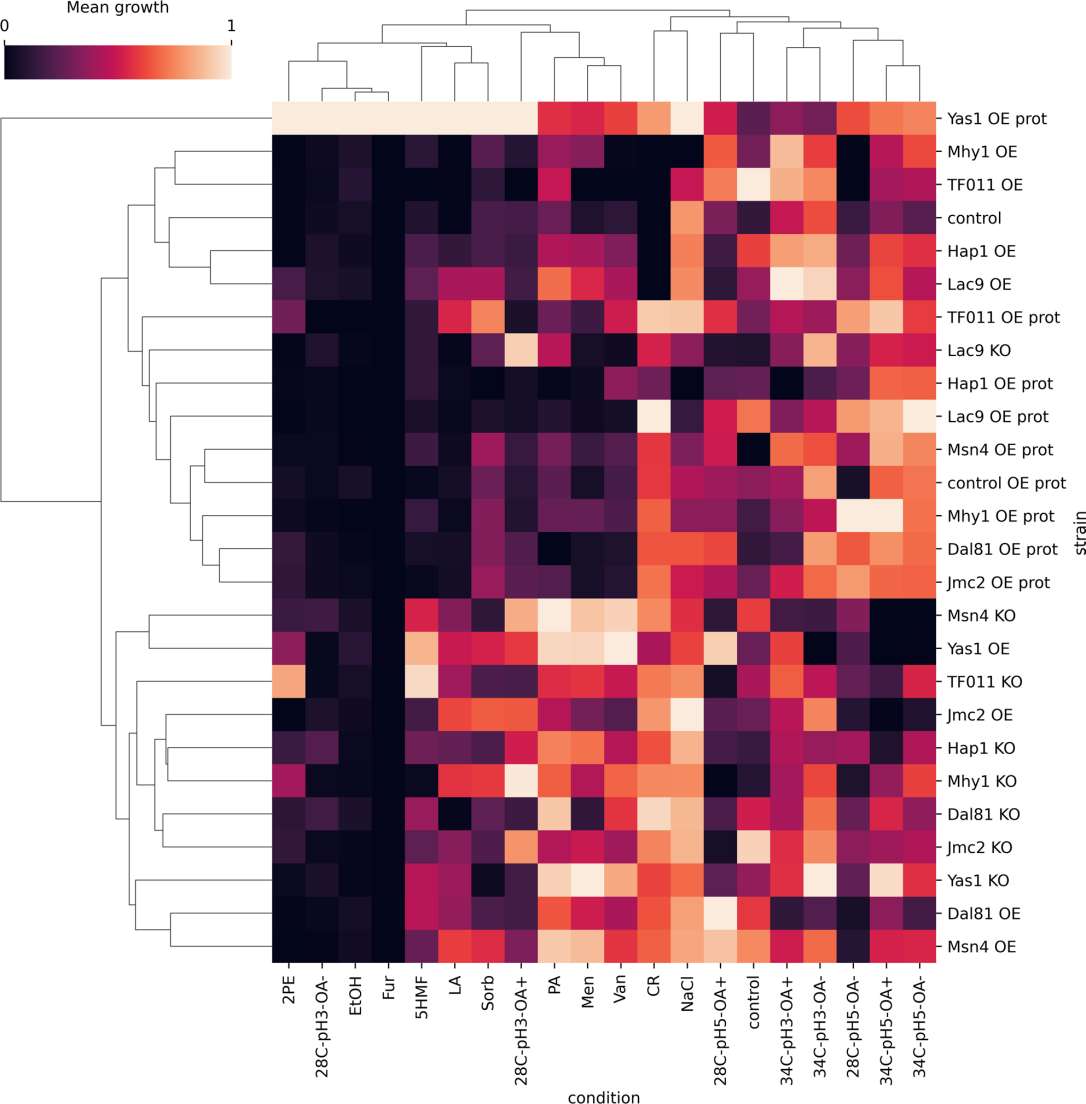
Clustered heatmap with a hierarchical dendrogram of normalized growth profiles of TF-modified and the control *Y. lipolytica* strains in process parameters and chemical stress factor-perturbed —data acquired at 48 h of culturing. All the data were normalized within each condition. X axis: treatment conditions; Y axis: TF-modified strains. Squares at the cross-points show the RC value for a specific strain and condition. Color code is explained in the legend

Summary and global clustering of the TF-engineering-driven responses across both the process parameters and chemical stress factor inflictions are presented in Fig. 7. Both control conditions were clustered together within a bigger subcluster represented mainly by thermal treatment (34 °C) conditions. The second major subcluster gathered most of the chemical-treatment conditions and the process parameters low pH condition. In this subcluster, similar phenotypes were observed in response to 2PE, pH3/OA-, EtOH, and Fur, as the most growth-limiting group, followed by 5HMF, LA, and Sorb, as the second most detrimental, and the pH3/OA+, PA, Men, Van, as the least inhibitory to the strains’ growth.

Considering an individual TF’s response, a single, own-cluster phenotype is represented by the Yas1-OE prot strain, displaying a unique resistance-promoting phenotype to both process parameters and chemical stress factors (Fig. 7). Any chemical stress, and individually inflicted pH 3–34 °C, awakened the growth-promoting phenotype. All the OE prot phenotypes were clustered together with the respective control strain, indicating the predominant (or combined) role of the rProt OE rather than the specific TF’s action. Lac9 KO globally displayed a similar phenotype to the OE prot, yet its unique character was observed in pH 3 cultures. Together with Msn4-KO, Mhy1-KO, and Jcm2-KO, Lac9-KO triggered a low pH-resistant phenotype.

Interestingly, Msn4-KO was clustered together with the Yas1-OE strain, while Yas1-KO was clustered with the Msn4-OE strain. The former cluster was characterized by high sensitivity to temperature upshift and high resistance to pH 3, PA, Men, Van, and 5HMF, combined with low-level but detectable growth under 2PE. The latter displayed a fairly temperature-resistant phenotype, and complete growth inhibition in the presence of 2PE and pH 3; demonstrating an inverted mode of action. In contrast, the OE and KO phenotypes by Mhy1 and TF011 were highly correlated. Of note, TF011- and Mhy1-KO contributed to resistance to 2PE toxicity and sensitivity to 34 °C. On the other hand, OE of Mhy1 and TF011 made the strains very sensitive to OA- (OA + vs. OA- pH 5, 28 °C) and CR, but rendered the strains relatively resistant to the lethal combination of 34 °C with pH 3. Dal81 OE caused thermal treatment sensitivity (vs. KO), higher resistance to oxidative stress (Men), and LA.

## Discussion

In this study, we phenotyped TF-related genotypes in *Y. lipolytica* across a range of industrially relevant stress conditions. Eight TFs were selected for this study based on previously conducted high-throughput screens of OE rProt genotypes [28], presented in YaliFunTome database. Here, two more genotypes were developed – OE and KO, to provide a comprehensive inspection of the TFs’ operation. The three genotypes per TF were then screened for growth under exposure to stressful conditions.

One of the most striking phenotypes was displayed by *Y. lipolytica* strains modified in the *YAS1 locus*. In *Y. lipolytica*, Yas1 was identified as a TF essential for cytochrome p450 induction in response to alkanes, operating as a heteromeric Yas1/Yas2 complex [48]. UniProt similarity search found *Y. lipolytica* Yas1 similar to *S. cerevisiae* Ino4 TF required for derepression of inositol-choline-regulated genes involved in phospholipid synthesis [49, 50]. By similarity to Yas1/Yas2 from *Y. lipolytica*, Ino4 forms a heterodimer with Ino2. Ino4 is essential for derepressing these genes when inositol is scarce, thereby controlling lipid metabolism and membrane synthesis.

As shown in the YaliFunTome database (link in Table 1), OE of Yas1 (in the background of OE prot) led to a high upshift in growth at pH 3.0, particularly notable at OA + and organic nitrogen presence. The lower the temperature (from 34 °C to 22 °C), the more pronounced the effect. The critical interplay between acidic pH and organic nitrogen sources for *Y. lipolytica* has been elegantly explained previously, using a *rim101* mutant [51]. Current results corroborate the previous conclusions. As illustrated in Fig. 1 (row Yas1, columns 1, 2) or Fig. 7, a combination of Yas1 OE and rProt OE triggers the remarkable “low pH-insensitive”. Yet, without the contribution of OE rProt, the pH-resistant phenotype was diminished (Figs. 2 and 3).

Moreover, the Yas1-OE strain was significantly temperature-sensitive (Fig. 1, row Yas1, columns 7, 8; Figs. 3B and 7), and the phenotype was inverted in the Yas1-KO strain (significantly promoted growth at 34 °C; Fig. 2). The background rProt overexpression buffered this direct effect (yielding positive RC under Yas1-OE). It is necessary to note that the sole OE of rProt enhanced growth under 34 °C, and, to our interpretation, this factor masked the actual effect of Yas1-OE. When not accompanied by rProt overexpression, Yas1 OE promotes growth under standard cultivation conditions (Figs. 1 and 2, and 3), and exerts a thermosensitive phenotype, directly inverted when KO. Under low pH, an opposite pattern is displayed – for Yas1-KO, growth is non-significantly limited, and improves when OE; but the remarkable “low pH-resistance” phenotype is mainly displayed under a combination of Yas1 and rProt OE.

**Fig. 8:**
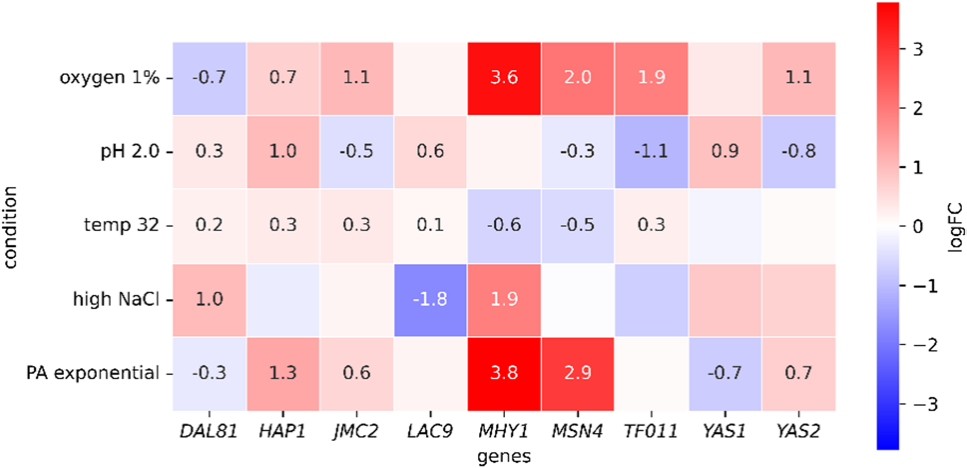
Differential gene expression analysis of RNA-seq datasets retrieved from public repository. The heatmap displays log2 fold-change (logFC) values of selected genes relative to their corresponding control conditions. Numeric annotations indicate logFC values for genes exhibiting statistically significant differential expression (adjusted P value < 0.05). RNA-seq data were obtained from the following BioProjects: oxygen 1% (PRJNA205557; control: oxygen 21%), pH 2.0 (PRJNA319797; control: pH 6.0), temperature 32 °C (PRJNA531619; control: standard conditions), high NaCl (PRJNA1196998; control: high glucose concentration), and PA (PRJNA955139; control: glucose). Conditions within the same BioProject are directly comparable, as each comparison refers to a shared project-specific control. Detailed dataset information is provided in Table S6

So the question is, what else is initiated due to rProt OE that assists the Yas1-driven low pH-insensitivity? Referring to our previous results [21], Yas1 was not deregulated due to OE of any of the biochemically different rProts studied there (intracellular, secreted, highly glycosylated, or highly disulfide-bonded). Under standard growth conditions, it also did not induce any specific phenotype in the OE rProt genotype, demonstrating that the phenotypes are condition-dependent (Fig. 2). It remains to be settled whether it is Yas1 itself or its dimeric partner responsive to temperature/low pH, or the phenotype that it elicits is sensitive/resistant to high temperature/low pH, or a combination of these and others. Regretfully, Yas2 (its dimeric partner) was not covered by the YaliFunTome database screens and the current phenotype studies. To gain further insight into the TFs’ mechanism of action, we run transcriptomics “data recycling” (Fig. 8; Table S6). The data from previous BioProjects deposited in public repositories were uniformly processed from the repository-withdrawn raw reads. Under extremely low pH (2.0) *YAS1* was upregulated, but *YAS2* was downregulated (Fig. 8). In our studies, the overrepresentation of Yas1 in the cell facilitated growth under pH 3 (more when combined with OE prot). In transcriptomics, the overrepresentation of the *YAS1* transcript was natively induced under very low pH. Thus, some very vague consistency could be inferred. However, the downregulation of *YAS2* under the same pH raises more questions rather than providing explanations. Exactly the same can be concluded when comparing transcriptomics data with phenotyping for YAS/Yas under the elevated temperature. At the transcriptional level, no significant change was observed (Fig. 8), and at the phenotyping level, a consistent temperature-sensitive phenotype emerged due to *YAS1*-OE, and a temperature-resistant phenotype when KO.

Considering the known role of Yas1/Yas2 in derepression of phospholipid synthesis [52, 53] it was interesting to test its impact on 2PE, EtOH, and osmolytes (Sorb, NaCl) resistance, known to strongly act on the plasma membrane. Strikingly, Yas1 and Lac9 were the only TFs studied here that enhanced *Y. lipolytica* resistance to 2PE when overexpressed (with the same tendency for EtOH). For the other TFs studied, the TF’s KO enhanced growth in 2PE-containing medium (for statistical significance, see Table S5).

Altogether, this study shows that Yas1 OE enhances *Y. lipolytica*’s resistance to 2PE (tendency to EtOH), Sorb, and low pH, but decreases resistance to high temperature and NaCl. In reference to Yas1’s known biological role in phospholipid synthesis, it is tempting to search for the underlying mechanism in the plasma membrane condition. Alcohols and elevated temperature are known to increase fluidity and permeability of the plasmalemma. Indirect effects of low pH and osmolytes on plasma membrane actions are also known – they both increase permeability, but the fluidity is typically decreased (due to lipid protonation and peroxidation, as well as hypertension). Based on this knowledge and current findings, a hypothesis about increased/decreased membrane fluidity due to Yas1-driven genetic intervention is one of the most relevant to test.

The global pattern of stress-resistance displayed by the Yas1-OE strain was similar to the one expressed by the Msn4-KO strain (Fig. 7). They were characterized by high sensitivity to temperature upshift and high resistance to pH 3, PA, Men, Van, and 5HMF, combined with growth under 2PE. In contrast, their corresponding reverse-engineering counterpart strains exhibited a temperature-resistant phenotype and growth inhibition in the presence of 2PE and pH 3. That was also reflected in the contradictory expression profile of *MSN4* and *YAS1* in the recycled transcriptomics data (Fig. 8). Yet, the complementarity was not perfect, and, depending on the adopted environmental perturbations, either both OE/KO strains of the reverse-engineering counterpart strains were clustered together (Fig. 7).

Msn4 belongs to the general stress response TFs. In *Y. lipolytica*, Msn4 was reported to promote tolerance to acid-induced stress, as its deletion led to growth defects at pH 3.0 [54]. Our results do not comply with the previous data: strains with Msn4-KO genotype displayed improved growth under pH 3 (Fig. 1, row Msn4, column 1 and 2; Fig. 2). Also, Msn4-KO did not cause growth limitation in the presence of LA (Fig. 4); though its OE triggered higher LA-resistance than the KO counterpart. Our results suggest that Msn4 is directly involved in Sorb-induced osmostress response, presenting an inverted phenotype when KO. Moreover, the Msn4-KO genotype provides resistance to the lignocellulosic biomass derivatives – 5HMF and Van (Figs. 4, 5A and 7). The recycled transcriptomics data (Fig. 8) showed that *MSN4* is significantly transcriptionally responsive to OA- and PA (upregulation), as well as in response to low pH and elevated temperature (downregulation). Current phenotyping does not align in terms of the response to OA (no effect in Fig. 2). However, Msn4-engineering modulated growth at low pH and temperature upshift. In our previous transcriptomics dataset [21, 55] Msn4 was upregulated upon the onset of unfolded protein response (UPR) in *Y. lipolytica*. UPR is known to be inherently coupled with oxidative stress, stemming directly from ER-localized oxidative folding [56, 57, 58]. Here, its role in oxidative stress response was highlighted by a rapid initial growth of the Msn4-OE strain in the presence of Men (Fig. 4), but the advantage was lost later in the cultivation (so not considered in the comparative and clustering analyses).

In summary, the global pattern of the Msn4 operation in *Y. lipolytica* is characterized by its prominent role in resistance to high temperature and osmostress induced by Sorb, and counteraction in low pH resistance, and 2PE, 5HMF, and Van exposure (improved in Msn4-KO). An indication of its rapid and transient action in oxidative stress (Men) and mild acid stress (LA) is also highlighted, which is consistent with the literature data.

The second general stress response TF in *Y. lipolytica* is Mhy1 (Msn2) [59]. Previously, it was mainly studied in the context of promoting or abolishing filamentation [54, 60,61] and its role in lipid biosynthesis [62]. It is known to be upregulated in the pH shift to alkaline [59] and in glucose [61]. In the recycled dataset, *MHY1* displays an expression pattern that is similar to *MSN4* (Fig. 8). They are both highly upregulated in response to PA and OA-, and downregulated in response to 32 °C. *MHY1* differs from *MSN4* by its strong upregulation in response to the salinity stress (Fig. 8). Previous extensive studies decoupled Mhy1 from stress resistance [54, 60], demonstrating that Mhy1-KO does not negatively impact growth in the presence of stress factors (i.e., high temperature, oxidative stress (H2O2), nitrogen or glucose starvation. The same was found in the present study (Figs. 2 and 5AB). Indeed, genotype Mhy1-KO did not impose higher sensitivity to any of the stress factors tested here. Moreover, the Mhy1-KO grew better in low pH and a combination of high temperature and OA- (Fig. 2). It also displayed better performance in the presence of CR, Men, Sorb, Van, LA, and 2PE. In line, *MHY1* OE triggered enhanced sensitivity to NaCl, Van, and a tendency to limited growth in the presence of LA and 2PE (Fig. 5A, B). Considering the major aim of this study, to identify stress-resistance-promoting TF-targeted genotypes, Mhy1-KO would be recommended for its dual gain – abolishing problematic filamentation and promoting multiple stress resistance.

In terms of process parameters stress response and selected chemical stress inducers, Hap1-modified *Y. lipolytica* strains displayed growth profiles similar to those driven by Msn4 (Fig. 1 – rows Hap1 and Msn4; and Figs. 2, 4 and 5A, B), but their expression profiles under low pH and 32 °C were inverted (Fig. 8). Literature states that the Hap1’s ortholog from *S. cerevisiae* is an oxygen-sensing TF, mediating respiratory metabolism and growth of the budding yeast [63]. Its OE triggered a marked increase in the rProt synthesis capacity in *S. cerevisiae* [64]. By protein sequence similarity, the same oxidative metabolism-promoting activity was expected from the *Y. lipolytica* ortholog. In sharp contrast, our previous studies showed that genotype Hap1-OE strongly increased sensitivity to the oxygen limitation (impaired growth), and did not promote growth under oxygen supply [28], as seen for *S. cerevisiae*. In the current study covering three genotypes (OE, KO, OE prot), a specific effect of OA could be observed for the Hap1-KO genotype (Fig. 1, row Hap1, columns 1 vs. 2, 7 vs. 8). When the strain was grown at pH 3.0, the lack of Hap1 promoted growth under OA-. Indeed, whenever Hap1 was absent (KO), the growth was at least slightly higher than the control under oxygen limitation (Fig. 1, condition: 34 °C/pH 3 not considered; Fig. 2). From the YaliFunTome phenotyping data (a link in Table 1), we concluded that Hap1 is involved in the low pH and high temperature responses. Indeed, pH was a significant factor describing the Hap1-OE rProt phenotype previously [28]. Now, with the KO strain, it is possible to see that under low pH, the absence of Hap1 promotes growth (Figs. 1, 2 and 3A); the effect is not observed at a pH of 5.0. Consistently, in YaliFunTome, Hap1-OE rProt displayed a dramatic decrease in growth under pH 3 (with organic nitrogen), presenting an inverted phenotype in response to pH. Moreover, the elevated temperature nicely separated the Hap1-engineered phenotypes: growth of Hap1-KO was abolished, while Hap1-OE(prot) exhibited significant thermotolerance (Figs. 1, 2 and 3); proving that the TF is indeed implicated in multiple stress responses. The expression profile highly responsive to stress treatments corroborates this statement (Fig. 8). In the recycled data, the temperature of 32 °C, low pH, and oxygen limitation were all associated with significant upregulation of *HAP1*, suggesting its direct role in stress response (Fig. 8). When *HAP1* was overrepresented, the strain always grew minimally below the control and clearly below the Hap1-KO. It is thus tempting to state that abundance of ylHap1 is a signal of stress occurrence, directly leading to growth cessation.

Several TFs covered by this study are underdescribed in the literature on *Y. lipolytica*, yet they elicited interesting phenotypes when genetically engineered. Lac9 is known as an inducer of genes involved in the utilization of alternative carbon sources (lactose/galactose). In the previous transcriptomics data, it was upregulated in response to low pH (Fig. 8). In our studies, Lac9-KO triggered a low pH-resistant phenotype (Fig. 2). Previously, the Lac9-OE prot strain grew slightly worse under pH 3 than in pH 5 or 7, and it was the “neutral pH” level that contributed significantly to the growth model represented by that strain. Current data are consistent with the previous results. Expression of such an inverted phenotype, and transcriptional upregulation in response to very low pH suggest that Lac9’s activity is modulated by the ambient pH, and contributes to growth limitation in response to low pH. When it is absent, growth under low pH can be maintained. Moreover, *LAC9* displayed a tremendous downregulation under salinity stress (Fig. 8). Here, high salinity (NaCl) triggered a significant phenotype for the Lac9-engineered strain as well (Fig. 5A, B), by strongly abolishing growth under Lac9 absence. Interestingly, the phenotype was specific to NaCl, and it was not displayed under Sorb treatment.

Based on similarity to *S. cerevisiae*, Dal81 is deemed to be involved in the use of non‑preferred nitrogen sources. Yet, in our high-throughput screens covering organic vs. inorganic sources, “nitrogen” was found to be a non-significant variable in driving growth [28]. The other putative function is glucose transport regulator (Rgt1-related), which is more consistent with our functional screens, as the term “carbon” was found significant, and glucose (vs glycerol) was the main level describing Dal81-OE prot phenotype. At the transcriptional level, Dal81 was shown to be slightly induced by elevated temperature and low pH (Fig. 8). Our results demonstrated that Dal81’s phenotype was relevant to thermal treatment only when combined with rProt synthesis (which was rather a result of the rProt biosynthesis). Any resistance to low pH was observed only in the Dal81-KO genotype. On the other hand, current data suggest its direct implication in Men-induced oxidative stress management (Fig. 5A, B). Strain Dal81-OE displayed a strong resistance to LA, while Dal81-KO was resistant to Fur, PA, and 2PE. Consistently, PA treatment triggered downregulation in *DAL81* expression (Fig. 8). A combination of transcriptomics and phenotyping data, strongly suggest Dal81’s direct role in salinity stress response. Yet, the direct mechanisms of its action remain to be settled.

Across the tests run in this research, we observed a specific behavior of strains with a background rProt synthesis, which could now be dissected from the non-burdened strains (in contrast to [28]). To our surprise, the synthesis of a small fluorescent protein contributed to the stress factor-specific advantage of *Y. lipolytica* cells. Most striking phenotypes were observed in response to elevated temperature (Fig. 1), where background rProt significantly improved stress resistance. Similar observations concern growth in CR, LA, and 2PE (Figs. 4 and 5B). Moreover, in several specific cases, a combined action of TF-engineering and the rProt synthesis could release the full beneficial potential of a given TF. Most striking examples are Yas1-OE prot resistance to pH 3, Lac9-, TF011-, Mhy1-, Jcm2-, and Dal81-OE prot under exposure to OA-, as well as TF011-, Jcm2-, Msn4-, Mhy1-, and Dal81-OE prot resistance to elevated temperature (with or without additional OA-). Likewise, several such beneficial combinations of TF and rProt were observed for chemical stress resistant phenotypes, including, extraordinarily advantageous phenotype Yas1-OE prot (the most resistant genotype, from the three analyzed, to CR, Sorb, 5HMF, Fur, LA, 2PE, EtOH, and uniquely to NaCl). Other examples cover Lac9-OE prot perturbed with CR, or TF011-OE prot exposed to Sorb. Obviously, some other components necessary for developing a highly resistant phenotype are awakened by rProt biosynthesis. Such observations are (only seemingly) contradictory to our previous results on the combined effects of rProt production-driven metabolic burden and costs, and exposure to environmental stresses [22, 65]. Based on those studies, we concluded that the extra burden of rProt synthesis makes *Y. lipolytica* cells more susceptible to stress (low pH and OA-). Here, we mainly see the opposite. However, previously we used strains severely burdened with the overproduction of two large, post-translationally modified and secreted rProts. The strain overproducing a small fluorescent protein (similarly burdened as the current OE prot strains) was a comparison partner, considered the non-burdened partner. Having a bigger picture now, we concluded that some background, mild internal stress in the form of rProt synthesis may be beneficial for the factor-specific stress resistance.

In summary, this study provides either new knowledge or a deeper understanding of selected TFs’ implication in process parameters and chemical stress resistance in *Y. lipolytica*. Based on the newly gained knowledge, global metabolic engineering strategies are proposed to enhance the robustness of *Y. lipolytica* under exposure to industrially relevant stress factors, like Msn4-, Hap1-OE, and Yas1-KO for improved thermotolerance, Yas1-OE for enhanced osmostress resistance, Dal81-OE, but TF011 and Mhy1-KO for insensitivity to LA, and Yas1-OE, but KO of TF011 and Mhy1 for significantly enhanced growth in the presence of highly valued aroma and antifungal compound – 2PE.

## Supplementary Information

Below is the link to the electronic supplementary material.

Supplementary Material 1.

## Data Availability

All the data are presented in this manuscript and the supplementary materials. Material will be made available upon direct request to EC.
