# Supplementary material for: Phenotyping transcription factors-related genotypes in Y. lipolytica across a range of industrially relevant process parameters and chemical stress conditions
Source: Microb Cell Fact. 2026 Mar 17;25:111. doi: 10.1186/s12934-026-02986-z (PMC13112681; doi:10.1186/s12934-026-02986-z)
Supplement: Supplementary file 1 — Supplementary Material 1. [file 12934_2026_2986_MOESM1_ESM.pdf]

Supplementary material

Phenotyping Transcription Factors-related genotypes in *Y. lipolytica* across a range of industrially relevant process parameters and chemical stress conditions

Maria Gorczyca[1], Abinaya M. G. Ponmalar[1], Julia Matz[1], Jean-Marc Nicaud[2], Ewelina Celińska[1*]

[1]Department of Biotechnology and Food Microbiology, Poznan University of Life Sciences, ul. Wojska Polskiego 48, 60-637 Poznań, Poland

[2]Université Paris-Saclay, INRAE, AgroParisTech, Micalis Institute, 78350 Jouy-en-Josas, France; Yalicolor, 48 rue Pablo Picasso, 78190 Trappes, France

*Corresponding author: ewelina.celinska@up.poznan.pl, ORCID: 0000-0001-8372-8459

Figure S1. The KO cassettes were constructed on the backbone of the pV2 vector [35]

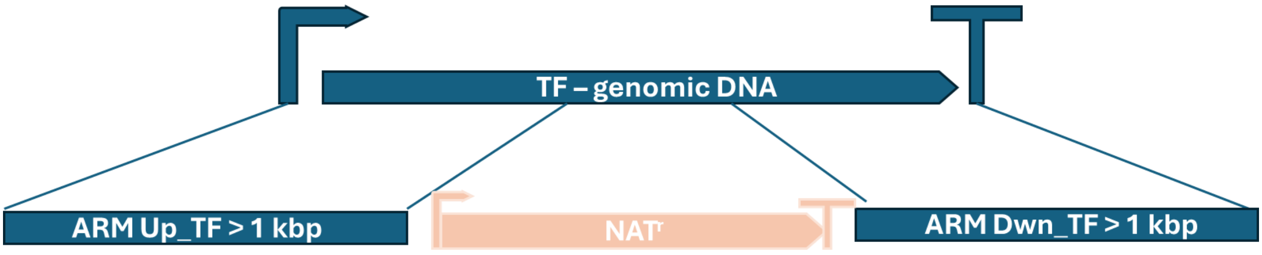

Table S1. List of *E. coli* strains used in this study.

ECB number	Parental strain (background)	Vector	Gene / modification
ECB_33	JM109	JMP62	JMP62-URA3
ECB_34	JM109	JMP62	JMP62-LEU2
ECB_194	DH5alpha	JMP62_Leu2ex-pTEF	HAP1_codon optimized, ER removed
ECB_196	DH5alpha	JMP62_Leu2ex-pTEF	LAC9_codon optimized, ER removed
ECB_197	DH5alpha	JMP62_Leu2ex-pTEF	TF011_codon optimized, ER removed
ECB_198	DH5alpha	JMP62_Leu2ex-pTEF	MHY1_codon optimized, ER removed
ECB_238	DH5alpha	JMP62-Leu2	YAS1
ECB_241	DH5alpha	JMP62-Leu2	MSN4
ECB_244	DH5alpha	JMP62-Leu2	DAL81
ECB_283	DH5alpha	JMP62-Leu2	JMC2
ECB_268	DH5alpha	pV2 (GGE140) EcoRI-NotI	ArmUP DAL81 - Nat - ArmDN DAL81
ECB_269	DH5alpha	pV2 (GGE140) EcoRI-NotI	ArmUP YAS1 - Nat - ArmDN YAS1
ECB_270	DH5alpha	pV2 (GGE140) EcoRI-NotI	ArmUP LAC9 - Nat - ArmDN LAC9
ECB_272	DH5alpha	pV2 (GGE140) EcoRI-NotI	ArmUP MSN4 - Nat - ArmDN MSN4
ECB_275	DH5alpha	pV2 (GGE140) EcoRI-NotI	ArmUP JMC2 - Nat - ArmDN JMC2
ECB_277	DH5alpha	pV2 (GGE140) EcoRI-NotI	ArmUP MHY1 - Nat - ArmDN MHY1
ECB_281	DH5alpha	pV2 (GGE140) EcoRI-NotI	ArmUP TF011 - Nat - ArmDN TF011
ECB_103	JM109	pV2-EcoRI NotI	ArmUP HAP1 - NATr- ArmDNn HAP1

Table S2. List of *Y. lipolytica* strains used in this study. All JMY2566-derivatives were created previously [1,2].

ECY number	Parental strain	modification	Phenotype
ECY_352	Po1f leu-ura-	pJMP62-ura3-empty + pJMP62-leu-empty	ura+ leu+ (control)
ECY_644	ECY596 empty ura+ leu-	JMP62 leu2-TF011	ura+TF011 leu+
ECY_647	ECY596 empty ura+ leu-	JMP62 leu2-MHY1	ura+Mhy1 leu+
ECY_649	ECY596 empty ura+ leu-	JMP62 leu2-Hap1	ura+Hap1 leu+
ECY_651	ECY596 empty ura+ leu-	JMP62 leu2-LAC9	ura+Lac9 leu+
ECY_656	ECY596 empty ura+ leu-	JMP62 leu2-DAL81	ura+Dal81 leu+
ECY_658	ECY596 empty ura+ leu-	JMP62 leu2-YAS1	ura+Yas1 leu+
ECY_667	ECY596 empty ura+ leu-	JMP62 leu2-MSN4	ura+ Msn4 leu+
ECY_995	ECY352 (Po1f (empty_ura3_empty_leu2)	KO HAP1 - HAP1-ArmUP-NATr-HAP1-ArmDN	KO Hap1
ECY_997	ECY352 (Po1f (empty_ura3_empty_leu2)	KO DAL81 - DAL81-ArmUP-NATr-DAL81-ArmDN	KO Dal81
ECY_1002	ECY352 (Po1f (empty_ura3_empty_leu2)	KO YAS1 - YAS1-ArmUP-NATr-YAS1-ArmDN	KO Yas1
ECY_1007	ECY352 (Po1f (empty_ura3_empty_leu2)	KO MSN4 - MSN4-ArmUP-NATr-MSN4-ArmDN	KO Msn4
ECY_1018	ECY596 (Po1f (empty_Ura3_leu-)	JMP62 leu2-JMC2	ura+ Jmc2 leu+
ECY_1040	ECY352 (Po1f (empty_ura3_empty_leu2)	KO MHY1 - MHY1-ArmUP-NATr-MHY1-ArmDN	KO Mhy1
ECY_1044	ECY352 (Po1f (empty_ura3_empty_leu2)	KO TF011 – TF011-ArmUP-NATr-TF011-ArmDN	KO TF011
ECY_1045	ECY352 (Po1f (empty_ura3_empty_leu2)	KO JMC2 - JMC2-ArmUP-NATr-JMC2-ArmDN	KO Jmc2
ECY_1050	ECY352 (Po1f (empty_ura3_empty_leu2)	KO LAC9 - LAC9-ArmUP-NATr-LAC9-ArmDN	KO Lac9
ECY_119	JMY2566 (MATa, ura3::pTEF-RedStar2-LEU2ex-Zeta, leu2-270, xpr2-322)	MATa, ura3::pTEF-RedStar2-LEU2ex-Zeta-URA3ex-pTEF, leu2-270, xpr2-322	FL+ (control rProt)
ECY_62	JMY2566	MATa, ura3::pTEF-RedStar2-LEU2ex-Zeta-URA3ex-pTEF-JMC2, leu2-270, xpr2-322	Jmc2 OE, FL+
ECY_64	JMY2566	MATa, ura3::pTEF-RedStar2-LEU2ex-Zeta-URA3ex-pTEF-TF011, leu2-270, xpr2-322	TF011 OE, FL+
ECY_89	JMY2566	MATa, ura3::pTEF-RedStar2-LEU2ex-Zeta-URA3ex-pTEF-LAC9, leu2-270, xpr2-322	Lac9 OE, FL+
ECY_136	JMY2566	MATa, ura3::pTEF-RedStar2-LEU2ex-Zeta-URA3ex-pTEF-YAS1, leu2-270, xpr2-322	Yas1 OE, FL+
ECY_148	JMY2566	MATa, ura3::pTEF-RedStar2-LEU2ex-Zeta-URA3ex-pTEF-MHY1, leu2-270, xpr2-322	Mhy1 OE, FL+
ECY_160	JMY2566	MATa, ura3::pTEF-RedStar2-LEU2ex-Zeta-URA3ex-pTEF-MSN4, leu2-270, xpr2-322	Msn4 OE, FL+
ECY_171	JMY2566	MATa, ura3::pTEF-RedStar2-LEU2ex-Zeta-URA3ex-pTEF-DAL81, leu2-270, xpr2-322	Dal81 OE, FL+
ECY_173	JMY2566	MATa, ura3::pTEF-RedStar2-LEU2ex-Zeta-URA3ex-pTEF-HAP1, leu2-270, xpr2-322	Hap1 OE, FL+

[1] Leplat, C., Nicaud, J.M., Rossignol, T., 2015. High-throughput transformation method for Yarrowia lipolytica mutant library screening. FEMS Yeast Res 15, 1–9. https://doi.org/10.1093/femsyr/fov052

[2] Leplat, C., Nicaud, J.-M.M., Rossignol, T., 2018. Overexpression screen reveals transcription factors involved in lipid accumulation in Yarrowia lipolytica. FEMS Yeast Res 18, 1–9. https://doi.org/10.1093/femsyr/foy037

Table S3. Codon-optimized, synthetic versions of TFs for OE without *BamHI* and *AvrII* restriction sites inside the sequence, with *BamHI* and *AvrII* overhangs.

Gene	Sequence
HAP1	AAGGATCCATGACCGACCAAGAGTCTCGACGAAACGGCCTCGAGCAGCTGGCCGTGCGAGCCATTGCCGAGATGCGAGAAGGCCAGGGACCTCGACCTC<br>CTGACACTCGAGATCAGCAGCCCCTGGACCTGCTGCCTGACCACGAGGACAAGTCTGTGAACAACAACGGCACCCGACTGGCTCCCTACGAGCAGTGCATC<br>CCCGACCTGTCTGCCCCTGGCGCCGGATTCGAGGACGACTTCTCTCGAGTGGTGAAGCGACGACGACGAGCCCCTGTGTCTTGCCTGCTGTGCCGAAAGCG<br>AAAGGTCCGATGCGACAAGCAGATGCCCTGCTCTGCCTGCAAGACCGCCAACGTGACCGGCTCTTGCGAGTACGCCCCTCCTACCTGGGGCGGACGAGAG<br>GTGCGAGGCGGCCAAGAGATCCAGTTCACCGTGGACGAGGCTGAGCCCATCTCTCAGCCTCCAGGCAACAACTCTTCTAACTCTGACACCCGAGGCCACCA<br>CTCCTCTTCGTCCTCTTGCTCTAACATCTCTGTGCCTCCAATGCAGAAGCCCCTGACACCTCCTCAGATGCTGCAGCCTATCGTGTCTCCTCTGCAGCCCACCG<br>TGAAGTCTAAGTCCTCTCACCAGCGAATGTACGGACGATTCCCCAACGCCGCCAACCTGCTGCGAGCTGACCTGTCCGAGCGACAGCGAGCCAAGGGCGTC<br>GAGGTGCCTCCTTCGCCTCGAAAGAAGCAGCCTCTGCACAACGACGCCAAGTGCAAGCGAGCTATTGAGACTCTGCAAGAAACCCGACGACGAAGATCTG<br>ACTCTCCTATCGACCCCGTGTACAAGCCCCTGCTGGATCTGTTCCCCGGCCAGCAGTACTGTGAGTTCCTGATCGACTCGTTCCTGAAGCGAGTGAACACCG<br>TGCACTACTGCGCTTGCCCCGAGGGCCTGCGAACCGACTTCAACGCCCTGTGGTCTGCCAAGCAGCGAATCGAGGACGGCGAGCAGTTCAACGCTCGAACC<br>TACCTGAACCTGCCTTCTATCGCCGTGATGCTGCTGGTGTTCCGACTCGGACGAGGATCTTACCCTCTGAAGGACTGGTCCCCTCCTAACTACTGCATCAAGG<br>ACGGCCACCGAGACAACACCTACCTCGGACCTCAGGTGCTCGAGGTGGCCGAGGCCTGCCTGAACGCCTCTCACATCTTCCGACACGGCGGCCTCAAGACC<br>ATTCAGGCCCTGGTGCTGATGAAGCTGGACTGCCTGTACGCTCCCGACTCTGAGTACTCTCCCGACGGCGTGGACTCTGTGAACCTGACCGGCGTGATCCT<br>GCAGCTGGCTCTGTCTCAGGGCCTGTACATGGACCCCGCCAACTTCGACACCCAGTCTACCTCTGTGCTGGCCTTCCAGGACTCTCTGCTGGTGCAGTCTCCC<br>AAGGCTCACCGACGATCTATGTCTGTGTCCTCTGCCGGCTCTGCCTCTCTGATCGAGCCCTCTTCGGCCAAGCTGTGGCGAATCCTGTACGGCTGCGCCCTG<br>ATCCTGGACGCCAAGCGAAACCTGGAAGTGGGCATTCCCCTGTCTCTGCCCCAGGGCGACTGCGACGTGTACTTCGACCACACCGACGACGGCGGAAAGA<br>CCGCCTCTAACGTGCTGGCTTTCCGACGAGCCATGCTGCGATTCTGTGCCCTGTCTGGCTGCGTGATGACCGAGACTTCTCGACCCGTGATGTACCGAAACG<br>AGCCTCTGATCCAGCGACTCGGCCAGGACCTGCACAAGTTCGGCGTCGACGAGGTCGCCTCTCTGGCCCAGCTGCGAGAGGAACTGGGAATCCCCAAGGA<br>CGACTCCCTGGCTTTTTCTTCTGACGACGACCACGCTGACGAGTCCGACACCTCTTCTGTGGCCTCTTCTGCCACCTCTGCTCTGCAGCTGATCCACCGATGCC<br>GAATCTACTGCCTGTGGTCCAAGCTCTGTGTGCACTACGAGATGGACACCTACGACGCCTCTGAGGAACCCCTGTACGGACACCGAGAGCTGTGCGTGCGA<br>CTGGGCATCGAGCAGGACCAGGCCGGCGTGCTGTCTCAGTACCGAGTGATTCTGCGAATGTCCTCGCTGATCACCCTGCTCGTGTGGGTCGCCCGACGAAG<br>AACCGCCTCTGTCGAGCTGGGAGCCCACTCTTGGTACATCGTGTCCCTGGTGCACTCTATGATGGGACTGCCCCTGACCGGTGTCATCACCTGTTGGTTCCG<br>AGTGGCCATCCTGGCCAAGGAAACCGGCTCTCTGAAGGGCACCCGATGGGACCCTGACTGGCTGTTTCGACTGATTACCCAGGGAATCGCCGTGCTGGGC<br>GACACCGACGTGGGCGACCGACAGATGCACGGCCTGTGCGTGTCTTTCCTGAAGAAGGCCCGATCTGACCTGGGCCGACTGCTGGCTAAGATGCCCGGTT<br>CTGACCCCGCCTACCTGTCCTCTCAGTTCGACGCCATCGACAAGCTGGCCGAGGTTGAGGTGCTGTCTGAGGAAGATGAGGGACTGTAACCTAGGTT
LAC9	AAGGATCCATGGACACCGAGCACGAGGCCAAGATGGCTGACCCCAAGCGAACCGTGCAGGCCTGTGACCTGTGCCGACAAAAGAAGTCTCGATGCTGCG<br>GCACCCGACCTACCTGCGCCAACTGCGTGCGATCTGGATCTTCTTGCGTGTACGCCGCTCAGGCCCGAAAGCGACGACGAACTAAGCTCGAGGTGATGGAA<br>GAGAAGCTGCAGATCATGTCTCAGGACGAGAACTGCGACGTGGCCGCTCGACAAGAGCTGCTCGAGTCTTTCGCCCGACGACTCGAGAAGAAGCGAAAG<br>ACCGAACGAATCGACTCTCAGGTGAACAACGTGGTGCCCCAGATGTTCGCCGGCATGGTGGCTACCCCTTCCGAGACTCCCACCGACGTGCAGACCACCAT<br>GAACGCCATGGTGACCCCTTCTACCACTCCTCCTGATCCTATTCAGGCCCAGCAAGCCCAGCAGGCCGTCGAGCCCATCATGGACGTGCAGCACATCACCCC<br>TGACACTCAGAACCTGGCCAACCACGACACCCACGACTCTCATGACACTCATGACTCCCACGATACCCACAACACCAACCATAACACCCTGACCTACTCTAAC<br>CCCTTCACCTACGACGACCCCATGGCCAACCGACCTGTGTTCTGCTCTACCTCTGACGCCGGCCTCCGATGGATCGAGACTCAGACCTGTGACGTGGGCTTC<br>CACAAGCGATACTCTCACCTGACCGAGTCTCTGTTCGAGACTGGCCTGAACTTCCTGCGAAACTGGCGACCTCCTGTGCTGCCCTCTCGACCCATCTGCATGA<br>ACGAGGACGTGCTGCGATTCCTGGCTCGAACCTTCCTCGAGGCTGCTAACCCCTACCTGGACATCGTGGACGACGTGGACGTCCTGCGAGTGTTCAACGTG<br>GTCATCGAGTCTGACAACGCCAAGCAGGACGAGTACTACCGAGATGGCCACGTGCAGCTGTACAACCTGTCTTGCCCTGAGTCTCTGCTGTTCCACTCTTGC<br>GTGCTGGTGTCTCTCGAGATCTTTATGGACGTGACCCGAAACAAGAACGCCGCCGAGCTGGCCGACACCATTCTGCCCCTCGAGAAGGCCACCGCCGTGCG<br>AGATCAGGTGTACATCAACGCCCAGCGAGACTACATGAAGCTGATCTCTGGCGGCGGAGGCCCCGAAGGCCTGGGCAACACCTCTGACACCTCTAACTACA<br>CCCAGCTGTCTCTGCAGGCTTGCCTGGTGCTGCTGACCTCTACCACCGCCTCCTCTGAGCCCCAGCTGATGGAACCCCTGATCACCACCTGTATGCGACTGTG<br>CTACCAGATGCGACTGCACCGAGAGGAATCTATCGGCTTCATGTCTCGACATCGAACCCTCCGAAACTGCTTCTGGGTGACCTATATGTTCGAGGCCACCTT<br>CCTGGGCCGACTGGCTCGACGACCTCTTGTCCGAGACTACGACATCTCCCTGCTGTACCCCGAGTCTACCCTGCAGGACCAGCACCACGGACCTTTCACTCC<br>CAAGATGCACTCTACCGCCAACACTCCCTGCAACTCTTGGCTGAACCCCTACATCCGACTGTGTCGACTCCAGTCGCGAGTGTGCGACGAACTGTACAACAA<br>CGTGTTCCCTCCAGACACTCGAGATCCCGTCGAGCTGCTGCACATTGCCTCTAAGCTGGACTCTGAGCTGCAGTCTTGGCGAGCCTCTGTGCCCCACGACCT<br>GGACTTCGAGGCCGAGTACGACGCTGTGCACCCCATCGTGCTCGAGGCCTACGCTCCCCTGCACGGCGTGCACATTCGACCCGTGAACATCCAGGGCCTGA<br>TCAACGAACGATGCTGCAACGCCTACCGATTCCGAGTGCGAAACCTGGGACTGACCCTGCTGAACTGCATGTACCTGCACGTCACCATCCTGATCCACAAGA<br>CCTTTTTCTGGTATCCCCGATGGGCTCTGCCCGTGAACCTGTCCTTCGTGCGACCCACCTTCGGCTCTTACGACGTCGACGCCACTGCTCAGGCTCTGAACCC<br>CGCCGAGTACTCTTCTAACGGCAACTACATCCCTCCTCACCGAAACGAACACTTCTCTGCCTCGTTCTCTCAGAACCGATCTTACACCCAGTCTAACCAGTCTA<br>CCGGACAGGCCTTCAACCTGCCTCACGTGGACATTCCCTCCAAGCGAGTGTTTGCCTCCAACTCTCTGGTGCGATCTTCTGCCCGAAAGATCTGTAACGCTCT<br>GACCCGAATGGATATGCGATACACCTCTTTTATCTGGATTTTCGCTTTCTTCCCCACCGTGGCCTTCGACGTCCTGTTCCAGACCATCATCCTGACTCCTCTGG<br>ACTCCACCACCTCTGCCGACCTGTCTCGAATGCGATTCTGCATCAACTTTATGAAGAAGCTGAAGAACTCCAACATCAAGACCTCTGAGCAGGTCTTTCTGCC<br>CATCTACGAACAGCTGCTGGCCTACGCCACCGGCTTCGTGTCTAACTGCCTGACCACCAACGACGACGACGTCCAGGACAAGGGCGTCGGCGGCGAGCTG<br>GGATTCTTCCCACAGGCCACCGGCGACTCTTACTGGGACGGCTCTACCGGCGAGATGCCCGAGTTCGTGGCCATGTCTAACACTGCTCCCGTGATGATGCCC<br>GGCGAGTCTTGGACCCACTCTGGCGACGAGAACCGAGTGGTCCGACGAGTGATGTAACCTAGGTT

TF011	AAGGATCCATGTACGCCGAGGACTACCCCTATCCTCGATCTCCCAAGGAAACCACCGACTCTTCCGCTGAGCCCGCCTCTGACCTGGCCTCTGTGTCTGCCG<br>CTCCTCCTGCCGACGGCAACCCCGAGGAAACTACCGCTCCTACCACCGCCACCTCTGAAGAGGACGACAAGAAGAAGAACAAGCGACGAAAGCACAAGAA<br>GTCTCGAAACGGCTGCTTCAACTGCAAGAAGCTGCGAATCAAGTGCGACGAGTCTCACCCCGTGTGCAAGAACTGCCACCAGCGACACAAGGAATGCGTG<br>TGGCCCACCGTGGCCAAGGCCAAGGACCGAGAAGGCAAGGCCTCTGAGCTGTCTCCCACCGAGCTGGCCGCCTCTGCTCTGATGCACTCTACCTCTAAGCA<br>GACCATTCGACCCACCTCTATGGCCCGAATGTTCGACGTGGACTTCGCCGACCTCGAGCTGCTGCGATTCTACGTCGAGCACACCTCGCCTCAGCTGACCAA<br>GTCTTGCGGCGACGCCGACTACTCTTGGGTGCGATCTATCCCCTCTATGACCATCGTGAACTCTGCCCTGTACAAGTGCGTGCTGACCGTCGCCTCTATCCAC<br>AAGGCCCACATGTACCTGCCTGAGAAGTACACCTACGACCACGAGTACGCCCGAGAGCGAAAGCGACTGCGAAAGCACCTCGAGAAGCGACGAGATCGA<br>GAGAACCCCGGCGTGCCCATCAAGCTGCCTCCACCTGACATGACCCGAGTGACCGGCTACCTGAAGCCTAACTCTCAGCTGCTCGAGAAGATCGTCAAGGC<br>CTTCACCGAGGCTCTGGCCGGACACCGACAGTCTCTGGTGACCCTGAACGCCTCTAACCAGGAATCTATCCTGTCTACCTCTGTGGTGATCTTCATGATCGCC<br>CTGGCTCTGGGCGAGCTGATTCCCCTGATCAACTTCGAAGGCGGAGCCGACGTGATCGGCGTCGGCCGAGGCGTGATCGAGCTGGTGTTCCAGCTGTCTG<br>CTAAGGACCGAATGGTGCTGTTCCCTCTGCCTCCTCCTATGCCTAACCGATCTTACCTGCCTAACGAGCCCGTGCTGTGGCAGCTGATCTCTACCATTCCTGA<br>CATGGGCCACCGACAGATGTGCTCTATGGAACTGCAGCAGCTGATTGAGCTGTACAACCTGGACGTGAAGCACCGAGGCCAGTCTCATCTGCCCGGCTGG<br>GCCACCTATATCTCTGGCGGCTTCCTGCAGGCTACCCGAGCCGGCGATCCCTACTGCCTGGTGATCCTGGGCCACTACTGCGCCTTCGCTCACATGTCTCACT<br>CGTTCTTTTGGCTGCGAGATCGACTGTCCGGTGACCTCGAGGCCATCGTGGACGTGCTGCCCGAGGAATTCCACCACTACCTCGAGTGGCCCCGATCTATCT<br>GCGGCCGATTCGACATGAACTACCAGGACCTGATCTCTGGCCGACTGCAGAAGCTCACCCTCGACGACAAGGATGAGAAGATTGAGCCCGCTCACTCTGAG<br>CTTGGCCCCGGACCTTCTCAAGAGGGCCACCAGCAGCACTACGACGGCCCCATGCTGGACCTGCTCCAGCAGCAACAGCAACAACAGCCCCAGCAGCCTAT<br>TCCTCAGGTGTCTCAACAGCAGCAGCTTGACCAGCTGGACCAACAACAACTGACCCAGGACCAGATGCTGCAGTCGCCTCCTGAGCGACGACAGTCTAACC<br>ACTCGCAGCTGGGCCTGTCTGACAACCAGGACCCTCACATTCAGCAGCACATCCTGCACCAGCAACAACAACAGCAGCAACAACAGCAACAGCAGCAGCAC<br>CAGCAGCAGCAACATCAACAGCAACAACACCAACAACAACAACAACACCAGCACCAACACCAGCAGCAGCAGCCTCAGCAGCACCATCAGCACCAGCAGC<br>CGCAGCAACCCCAGCACTCTGGCGACCTGCACCACTCCGACCTGCCACCTCCGCCTGAGCAGCAGATCCCCGACATGGGCGACATGTACCAGTACTCTAACC<br>CCGGTCCTTCTGGCCACGTGGACCACCACTCGCTGAAGATGGACCACAACTCTTCTACCCACGGCTCTATGCTGTCTTACGGCCCCTAACCTAGGTT
MHY1	AAGGATCCATGGACCTGGAACTCGAGATCCCCGTGCTGCACTCTATGGACTCTCACCACCAGGTGGTCGACTCCCACCGACTGGCCCAGCAGCAGTTCCAG<br>TACCAGCAGATCCACATGCTCCAGCAGACCCTGTCTCAGCAGTACCCTCACACTCCCTCTACCACTCCTCCAATCTACATGCTGTCTCCCGCCGACTACGAGA<br>AGGACGCCGTGTCTATCTCTCCCGTGATGCTGTGGCCTCCTTCGGCTCACTCTCAGGCCTCTTACCACTACGAGATGCCCTCTGTGATCTCTCCCTCTCCTTCT<br>CCTACTCGATCTTTCTGCAACCCTCGAGAGCTGGAAGTGCAGGACGAGCTGGAACAGCTCGAGCAGCAGCCCGCTGCTCTGTCTGTCGAGCACCTGTTCGA<br>CATCGAGAACTCTTCTATCGAGTACGCCCACGACGAGCTGCACGACACCTCTTCGTGCTCTGACTCTCAGTCGTCTTTCTCGCCCCAGCAGTCTCCCGCTTCTC<br>CCGCCTCTACCTACTCGCCCCTCGAGGACGAGTTCCTGAACCTGGCCGGCTCTGAGCTGAAGTCTGAGCCCTCTGCTGACGACGAAAAGGACGACGTGGAC<br>ACCGAGCTGCCTCAGCAGCCTGAGATCATCATCCCCGTGTCTTGCCGAGGACGAAAGCCCTCTATCGACGACTCTAAGAAGACCTTCGTCTGCACCCACTGC<br>CAGCGACGATTCCGACGACAAGAGCACCTGAAGCGACACTTCCGATCTCTGCACACCCGAGAGAAGCCCTTCAACTGCGACACCTGTGGCAAGAAGTTCTC<br>TCGATCTGACAACCTGGCTCAGCACATGCGAACCCATCCTCGAGACTAACCTAGGTT

Table S4. Primers used in this study

Primers for TF amplification from genomic DNA for OE, with *BamHI* and *AvrII* overhangs.	
Primer	Sequence 5' → 3'
Msn4_BamHI_F	aggatccATGACCACCTACGATCTCAAC
Msn4_AvrII_R	acctaggTTAACATCGCCGCTTCTGTCG
Jmc2_BamHI_F	aggatccATGAAGAAGACGCTCAATCTCAAAC
Jmc2_AvrII_R	acctaggTCAGGCAGGGATCTTGGCAAGCAC
Dal81_BamHI_F	aggatccATGAAAAACGAATTCTTCGACC
Dal81_AvrII_R	acctaggTCACTGATTCATCTCCGAAACC
Yas1_BamHI_F	aggatccATGGATTCCCGATCAGCCTCC
Yas1_AvrII_R	acctaggCTAGACCGGAGACTCCTCTTTC
Primers for TFs' homologous arms amplification from genomic DNA for KO.	
Primer	Sequence 5' → 3'
ArmUp_Msn4_EcoRI_F	agaattcTGTATTAGGGTTGGGGTTAGGG
ArmUp_Msn4_KpnI_R	aggtaccGCCCTTGACAAACGACTGGGGC
ArmDn_Msn4_BamHI_F	aggatccTGCGACAAGCGGTTCAAGCGCC
ArmDn_Msn4_NotI_R	agcggccgcTGGCTGGAGTTGACTATCAGCG
ArmUP_Mhy1_EcoRI_F	aGAATTCGTGGTGTTGAAAAGGTGAAAAAGAG
ArmUp_Mhy1_KpnI_R	aGGTACCGGATTACAGAAGGATCTAGTGGGAG
ArmDn_Mhy1_BamHI_F	aGGATCCGCAGCCCGAGATCATCATCCC
ArmDn_Mhy1_NotI_R	aGCGGCCGCACACACTGCCGGGGGTATTTCT
ArmUp_Jmc2_KpnI_R	aGGTACCATCCTCATCTCCATTTACATCC
ArmUp_Jmc2_EcoRI_F	aGAATTCCATCCATAGATGTACGTTGCCC
ArmDn_Jmc2_BamHI_F	aGGATCCTGCCCTTCAGATGGGTCTCCCC
ArmDn_Jmc2_NotI_R	aGCGGCCGCTTTGTTCGCAACAGAGATTGCG
ArmUp_Dal81_EcoRI_F	aGAATTCCGCGACTCAGAACGGTGGAATC
ArmUp_Dal81_KpnI_R	aGGTACCAATATGGTCCAGGGTGGCGGTG
ArmDn_Dal81_BamHI_F	aGGATCCACTTCCGTATCGTTCATCCGGC
ArmDn_Dal81_NotI_R	aGCGGCCGCGACTTCTCGAATCGTTTGTGTC
ArmUp_Yas1_EcoRI_F	aGAATTCAGCACAGCCATGAAGCACTCTG
ArmUp_Yas1_KpnI_R	aGGTACCTGCTCGCTGGCAATGTGGTTGG
ArmDn_Yas1_BamHI_F	aGGATCCGCGGAGGGCAAGGAGCTCGAGC
ArmDn_Yas1_NotI_R	aGCGGCCGCCGCCGCAGCTACAGTTGGCGCC
ArmUp_TF11_EcoRI_F	aGAATTCGGTTGGCATGCACGATTTTGGC
ArmUp_TF11_KpnI_R	aGGTACCATGGACGGGATGGACCGCACCC
ArmDn_TF11_BamHI_F	aGGATCCGTCACTAGTGACCCTCAACGCC
ArmDn_TF11_NotI_R	aGCGGCCGCGGTTGTCGGAGAGTCCCAGTTG
ArmUp_Hap1_EcoRI_F	aaGAATTCCGGCCTGCAAGACTGCTAAC
ArmUp_Hap1_KpnI_R	aaGGTACCATGTACAGACCCTGCGAGAG
ArmDn_Hap1_BamHI_F	aaGGATCCGTTGTCGTTACCTCAGGGCG
ArmDn_Hap1_NotI_R	aaGCGGCCGCGGCATCTTGGCCAGCAAACG
ArmUp_Lac9_EcoRI_F	aGAATTCCACGACGCTGTCGATACCACCC
ArmUp_Lac9_KpnI_R	aGGTACCGCTGTGGAAAAGCAGAGACTCG
ArmDn_Lac9_BamHI_F	aGGATCCACCCAAGATGCACTCCACTGCC
ArmDn_Lac9_NotI_R	aGCGGCCGCTGAGTCTCCTGTAGCTTGAGGG

Table S5. Results of statistical tests for “inverted phenotypes” mentioned in the main text.

group1	group2	meandiff	p-adj	lower	upper	reject	condition	time
Yas1_KO	Yas1_OE	-22.67	0.0000	-27.29	-18.04	TRUE	34C-pH5-OA+	48
Yas1_KO	Yas1_OE	-2.06	0.0000	-2.98	-1.13	TRUE	34C-pH5-OA-	48
Yas1_OE	Yas1_OE_prot	2.72	0.0000	1.80	3.65	TRUE	34C-pH5-OA-	48
Jmc2_OE	Jmc2_OE_prot	2.15	0.0000	1.23	3.08	TRUE	34C-pH5-OA-	48
Jmc2_OE	Jmc2_OE_prot	15.84	0.0000	11.22	20.47	TRUE	34C-pH5-OA+	48
Yas1_OE	Yas1_OE_prot	16.96	0.0000	12.34	21.59	TRUE	34C-pH5-OA+	48
Dal81_KO	Dal81_OE	14.90	0.0000	9.58	20.22	TRUE	28C-pH5-OA+	48
TF011_KO	TF011_OE	12.57	0.0000	7.25	17.89	TRUE	28C-pH5-OA+	48
Mhy1_KO	Mhy1_OE	12.16	0.0000	6.84	17.48	TRUE	28C-pH5-OA+	48
Msn4_KO	Msn4_OE	14.33	0.0000	8.59	20.08	TRUE	28C-pH5-OA+	48
Yas1_KO	Yas1_OE	12.66	0.0000	6.91	18.40	TRUE	28C-pH5-OA+	48
Lac9_KO	Lac9_OE	0.07	0.0000	0.03	0.11	TRUE	Men	48
Dal81_KO	Dal81_OE	0.05	0.0002	0.02	0.09	TRUE	Men	48
TF011_KO	TF011_OE	-0.08	0.0000	-0.12	-0.04	TRUE	Men	48
Dal81_KO	Dal81_OE	-4.97	0.0000	-6.55	-3.39	TRUE	2PE	72
Hap1_KO	Hap1_OE	-1.32	0.0004	-2.27	-0.36	TRUE	2PE	48
Jmc2_KO	Jmc2_OE	-1.13	0.0055	-2.09	-0.17	TRUE	2PE	48
Lac9_KO	Lac9_OE	1.61	0.0000	0.65	2.57	TRUE	2PE	48
Mhy1_KO	Mhy1_OE	-3.56	0.0000	-4.52	-2.60	TRUE	2PE	48
Msn4_KO	Msn4_OE	-1.32	0.0003	-2.28	-0.37	TRUE	2PE	48
TF011_KO	TF011_OE	-7.08	0.0000	-8.12	-6.04	TRUE	2PE	48
Yas1_KO	Yas1_OE	2.87	0.0000	1.91	3.82	TRUE	2PE	48
Yas1_KO	Yas1_OE	1.84	0.0000	1.39	2.28	TRUE	Sorb	48
Msn4_KO	Msn4_OE	-1.32	0.0003	-2.28	-0.37	TRUE	2PE	48
Mhy1_KO	Mhy1_OE	-3.56	0.0000	-4.52	-2.60	TRUE	2PE	48
Jmc2_KO	Jmc2_OE	-1.13	0.0055	-2.09	-0.17	TRUE	2PE	48
Msn4_KO	Msn4_OE	1.62	0.0000	1.14	2.10	TRUE	Sorb	48
Lac9_KO	Lac9_OE	-0.77	0.0000	-1.19	-0.35	TRUE	28C-pH3-OA+	48

Table S6. BioProjects and their corresponding runs re-processed in this study. Controls relative to each BioProject are marked with an asterisk.

BioProject	Background strain	Run	Condition
PRJNA205557	JMY2900	SRR868666	*oxygen 21%
		SRR868667	*oxygen 21%
		SRR868668	*oxygen 21%
		SRR868669	*oxygen 21%
		SRR868670	*oxygen 21%
		SRR868671	oxygen 1%
		SRR868672	oxygen 1%
		SRR868673	oxygen 1%
		SRR868674	oxygen 1%
PRJNA319797	W29	SRR3457619	bioreactor pH 2.0
		SRR3457620	bioreactor pH 2.0
		SRR3457621	bioreactor pH 2.0
		SRR3457625	*bioreactor pH 6.0
		SRR3457626	*bioreactor pH 6.0
		SRR3457627	*bioreactor pH 6.0
PRJNA531619	W29	SRR8933517	chemostat temp 32
		SRR8933518	chemostat temp 32
		SRR8933519	chemostat temp 32
		SRR8933526	*chemostat standard conditions
		SRR8933527	*chemostat standard conditions
		SRR8933528	*chemostat standard conditions
		SRR8933529	*chemostat standard conditions
PRJNA955139	EXF-17398	SRR26275642	*glucose
		SRR26275643	*glucose
		SRR26275644	*glucose
		SRR26275653	propionic acid
		SRR26275654	propionic acid
		SRR26275655	propionic acid
PRJNA1196998	CA20	SRR31688493	YPD with high NaCl concentration
		SRR31688494	YPD with high NaCl concentration
		SRR31688495	YPD with high NaCl concentration
		SRR31688499	*YPD with high glucose concentration
		SRR31688505	*YPD with high glucose concentration
		SRR31688506	*YPD with high glucose concentration
